# Elasticity‐Driven Periodic Polarization Patterns in a Chiral Ferroelectric Nematic Fluid

**DOI:** 10.1002/adma.75002

**Published:** 2026-09-23

**Authors:** Anej Sterle, Peter Medle Rupnik, Luka Cmok, Aitor Erkoreka, Marta Lavrič, Natan Osterman, Calum J. Gibb, Jordan Hobbs, Josu Martinez‐Perdiguero, Richard J. Mandle, Alenka Mertelj, Nerea Sebastián

**Affiliations:** ^1^ Jožef Stefan Institute Ljubljana Slovenia; ^2^ Faculty of Mathematics and Physics University of Ljubljana Ljubljana Slovenia; ^3^ CENN Nanocenter Ljubljana Slovenia; ^4^ Department of Physics Faculty of Science and Technology University of the Basque Country UPV/EHU Bilbao Spain; ^5^ School of Chemistry University of Leeds Leeds UK; ^6^ School of Physics and Astronomy University of Leeds Leeds UK

**Keywords:** confinement effects, elastic instabilities, ferroelectric nematic fluids, polarization modulations, second harmonic generation

## Abstract

Ferroelectric nematic phases are a new class of polar fluids in which spontaneous polarization is directly coupled to the orientational order, providing unique opportunities for creating self‐organized materials with spatially modulated electric polarization and nonlinear optical response. Here we report the spontaneous emergence of polarization‐modulated textures in a chiral ferroelectric nematic material close to the transition to the chiral twist‐bend ferroelectric nematic phase. By systematically varying cell thickness and surface anchoring conditions, we map the formation of these modulated states, revealing stripe, square, and hexagonal morphologies determined via confinement conditions. These structures are directly translated into periodic modulation of the nonlinear optical response, as evidenced by second harmonic generation imaging. Comparison with an elasticity‐based theoretical framework and numerical free energy minimization shows that the instability originates from the softening of the bend elastic constant in the chiral nematic phase as the system approaches the lower‐temperature heliconical polar phase. The resulting elastic frustration, combined with confinement, drives the formation of spatially periodic director distortions, highlighting ferroelectric nematic fluids as a promising platform for self‐assembled nonlinear optical materials.

## Graphical abstract

Chiral polar ferroelectric nematic fluids exhibit spontaneous formation of stripe, square, and hexagonal polarization textures under confinement. Controlled by thickness and anchoring, these patterns produce spatially modulated nonlinear optical responses. Elastic softening near the twist‐bend transition drives instability, enabling self‐assembled, tunable architectures for advanced nonlinear optical materials. Polarization‐modulated textures spontaneously emerge in a chiral ferroelectric nematic near the transition to the polar heliconical phase. Systematic control of thickness and anchoring reveals stripe, square, and hexagonal morphologies. Second‐harmonic imaging confirms periodic nonlinear response. Elasticity theory and free‐energy minimization attribute the instability to bend elastic softening and confinement‐induced director distortions.

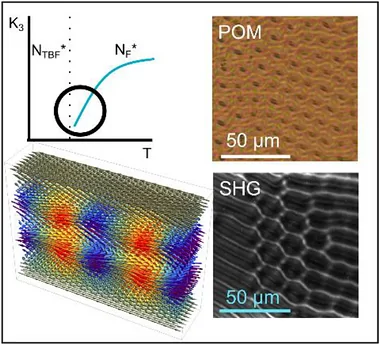

## 1 Introduction

Self‐assembly in soft matter provides a powerful route to functional materials with complex, hierarchically organized structures. Different mechanisms operate over different interaction ranges, resulting in a broad range of self‐assembly length scales, from the nanoscales, like in DNA [1], micelles, and block copolymers [2, 3], to the microscales, for example, in liquid crystals [4]. The latter are particularly attractive due to their intrinsic birefringence and strong responsiveness to external stimuli, enabling the formation of hierarchical optical architectures with potential for advanced photonic technologies [5, 6, 7, 8].

Chiral nematic liquid crystals (N*) form a self‐organized helical structure, in which the average molecular orientation, that is, the director ***n***, twists periodically, with the distance for a full 360° rotation defined as the pitch, *p*. These systems exhibit a variety of instabilities under different conditions, such as the well‐known Helfrich–Hurault periodic pseudolayer undulations under applied electric or magnetic fields [9, 10, 11, 12] as well as driven via geometric frustration [13, 14, 15], finger‐like instabilities via surface anchoring [14] or light driven 3D manipulation of the helical axis promoting 1D and 2D structures [16]. Heliconical instabilities under electric or magnetic fields in N* systems in which the bend elastic constant is lower than the twist (*K*_3_ < *K*_2_), were predicted by Gennes [17] and Meyer [18]. The early 2010s discovery of the twist‐bend nematic phase (N_TB_) in odd flexible dimers [19, 20, 21] featuring low values of *K*_3_, enabled experimental realization [22, 23]. In the N_TB_ phase, the director spontaneously forms a nanoscale heliconical structure, continuously processing around a fixed axis with a constant cone angle. In designed chiral N_TB_* systems, it was subsequently shown that geometric confinement frustration arising from the combination of thickness and surface alignment constraints acts as an elastic field, spontaneously forming undulation instabilities with different directions and dimensionalities [24, 25, 26, 27].

In recent years, research on liquid crystalline phases has undergone a profound transformation, with the discovery of the ferroelectric nematic counterparts: the ferroelectric nematic phase (N_F_) [28, 29, 30, 31] or the chiral ferroelectric nematic phase (N_F_*) [32, 33, 34]. In contrast to the apolar conventional nematic phase, these ferroelectric nematic phases are polar, exhibiting a spontaneous macroscopic polarization that is spatially uniform along the director in N_F_ and helically twisted in N_F_* [28, 29, 30, 31, 32, 33, 34]. Self‐assembled periodic patterns in polar nematic fluids, which exhibit large nonlinear optical coefficients [35, 36], are of interest both from a fundamental perspective, providing an experimentally accessible platform for exploring polarization topology, and for intrinsic regular modulation of nonlinear susceptibilities in reconfigurable materials. Recently, a novel heliconical twist‐bend ferroelectric nematic phase (N_TBF_) has been identified (Figure 1b) [37]. The same mesophase structure was identified for a system of hard‐rod polar molecular motives [38], tagged in this case as ^HC^N_F_, and in systems with the additional presence of cybotactic smectic correlations, addressed as HEC mesophase [39]. Similarly, a tilted layered phase with heliconical polarization, termed SmC^P^_H_, has also been reported [40], where the tilt direction processes about a helix orthogonal to the layer normal. All of them share a heliconical and polar molecular arrangement, making them both ferroelectric and chiral, with a periodicity in the range of visible light wavelengths.

**FIGURE 1:**
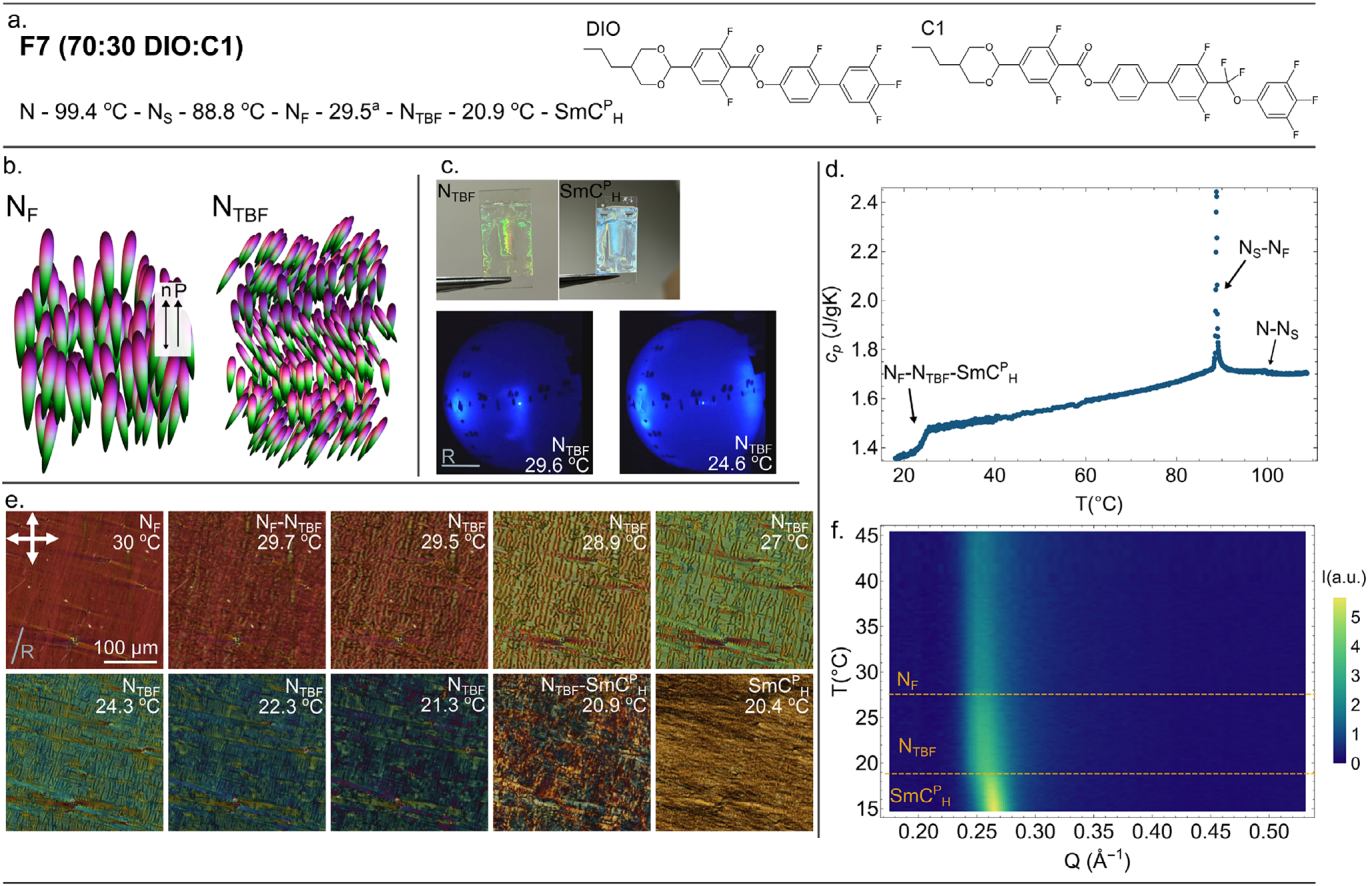
Room‐temperature ferroelectric twist‐bend nematic material. (a) F7 binary mixture phase behavior on cooling and chemical structure of the mixture components DIO and C1. (b) Schematic representation of the N_F_ and N_TBF_ phases. (c) Visible reflected light perpendicularly to the rubbing direction and light diffraction experiment at the onset of the N_TBF_ phase on 2 µm parallel‐rubbed cell, illuminated from behind and detected in a spherical screen. (d) Temperature profile of the heat capacity, *c*_*p*_(*T*), upon cooling. (e) N_F_–N_TBF_–SmC^P^_H_ phase sequence as seen by polarizing optical microscopy (POM) images on cooling in a 5 µm parallel‐rubbed cell. Double‐headed arrows indicate the direction of crossed polarizers, and the light‐blue line marked with R corresponds to the cell‐rubbing direction. (f) Temperature evolution of the X‐ray diffractograms obtained by integration of the 2D broad‐angle XRD for an unaligned sample in the vicinity of the N_F_–N_TBF_–SmC^P^_H_ phase transitions.

Here, we design a chiral ferroelectric material showing a room temperature N*_TBF_ phase and demonstrate the spontaneous formation of elasticity‐driven modulated structures, occurring in the vicinity of the transition from the N_F_* to the N_TBF_* phase. The appearance of these modulated polarization architectures is controlled and investigated as a function of confining thicknesses and surface anchoring geometry. By comparison with an elasticity‐driven model and numerical free‐energy minimization, we show that these instabilities arise from the combined effect of bend elastic constant softening in the nematic phase and confinement conditions as the system approaches the lower‐temperature N_TBF_* phase. The calculated elasticity‐driven polarization self‐assembled structures show low electrostatic cost, with electrostatic self‐interaction not playing a dominant role, highlighting an avenue for effective geometric control of 3D polarization patterning.

## 2 Results and Discussion

### 2.1 Material and Mesophase Behavior

To design our chiral material, we selected the binary mixture F7, composed of 70% DIO [28] and 30% Compound 1 (C1)(Figure 1), based on the phase diagram reported in [40], where C1 was originally introduced. This choice was made in order to stabilize the N_F_ phase over an extended temperature range. Motivated by the observed behavior, we subsequently revisited the phase behavior of the parent achiral mixture F7. This analysis led to the identification of a previously unresolved narrow temperature window corresponding to the N_TBF_ phase (Figure 1b), located between the N_F_ and SmC^P^_H_ phases originally reported. The heat–capacity profile obtained by high‐resolution AC calorimetry on cooling is shown in Figure 1d. The nematic–antiferroelectric nematic (N–N_S_, 99.4°C) and the antiferroelectric nematic to ferroelectric nematic (N_S_–N_F_, 88.8°C) phase transitions exhibit single and sharp peaks, demonstrating an excellent miscibility of both components. The negative peaks of the phase shift for both transitions (Figure S2) indicate second‐order phase transitions, in contrast to pure DIO, where both are weakly first order [41]. Phase assignment of these three mesophases is supported by SAXS, POM, SHG, and spontaneous polarization measurements (Figures S3 and S4), revealing a ferroelectric nematic phase extending from 88.8°C to almost room temperature.

Around 29°C, a clear textural transition is observed by POM (Figure 1e, Movie S1), accompanied by visible light diffraction along the rubbing direction (Figure 1c), suggesting the emergence of a periodic structure, with a characteristic length scale below the spatial resolution of POM. Using a 448 nm source, broad first‐order diffraction maxima emerge centered around 40 deg (with respect to the direct beam) at the transition and rapidly shifts to higher angles, suggesting an initial periodicity of the order of ∼700 nm (Figure 1c, see Experimental Section). The width of the peaks indicates that the periodic structure is not a perfect grating, but rather has a finite correlation length. X‐ray experiments confirm a nematic pattern (Figure 1f), identifying the mesophase as N_TBF_. On further cooling, a transition at 21°C (Figure 1e) shows a grainy uniform texture with SAXS revealing a narrowed shifted peak (Figures 1f and S3), indicating a transition to a tilted smectic phase. At lower temperatures, calorimetry shows a broad anomaly with long *c_p_* pretransitional wings. Although two separate transitions cannot be distinguished reliably (i.e., as two separate peaks) as in the case of POM, the possibility of two broad transitions occurring close to each other cannot be excluded from the *c_p_* profile either. The tilted smectic phase additionally exhibits selective reflection (Figure 1c), spontaneous polarization, as well as SHG signal (Figure S4). Thus, the mesophase is assigned to be a tilted smectic polar heliconical phase, SmC^P^_H_. Further analysis of the mesophases is beyond the scope of this manuscript, and here we will focus on the N_F_–N_TBF_ phase transition in the F7 chiral counterpart.

Building on the F7 mixture, we introduce chirality by adding 1 wt% of the chiral dopant S811 (Merck) to F7. F7*exhibits a chiral nematic (N*), chiral antiferroelectric N_S_*, chiral ferroelectric nematic (N_F_*), and a chiral N_TBF_*structure. The heat–capacity profile obtained by high‐resolution AC calorimetry on cooling shows that, compared to F7, the two high temperature phase transitions are shifted only slightly to lower temperatures, while their thermodynamic character remains unchanged (Figure S2). The chiral pitch *p* was evaluated via POM on cooling from the N* phase in a Grandjean–Cano (GC) wedge cell (EHC) with wedge angle θ = 0.68°, minimum thickness approximately 1.6 µm and parallel rubbing along the wedge direction. Figure 2a shows GC textures at different temperatures (full sequence in Movie S2). In the N* phase, the nonzero thickness in the thin edge promotes the π‐twist state, so GC regions start with this configuration. With increasing thickness, the structure adapts to the confinement creating a series of parallel line defects perpendicular to the wedge at a distance *L*, separating GC regions of different numbers of director twists. Within the observed thickness range, only thin dislocations are observed [42, 43], through which the structure twist jumps by π. Cooling into the N_S_* phase, the edge dislocations shift slightly toward thicker regions, but are preserved. After the strong destabilization at the transition to the N_F_*, the structure relaxes back into GC twist regions, now separated by edge dislocations accommodating 2π structure changes. This behavior has been well understood [32, 33], considering that in the N_F_* phase, the rubbed surface layer also determines the direction of polarization ***P*** [44, 45], limiting to structures with an even number of π‐twists under parallel rubbing. Accordingly, pitch is determined by *p*  =  2*L*tanθ in the N* and N_S_* phases and *p*  =  *Lt*anθ in the N_F_* phase, respectively (Figure 2b). While its behavior in the N* and N_S_* phases resembles that of the parent material DIO [43], it behaves differently in the N_F_* phase, showing a nearly constant *p* over more than 40°, while that of DIO (for similar doping characteristics) shows a pronounced linear increase on cooling.

**FIGURE 2:**
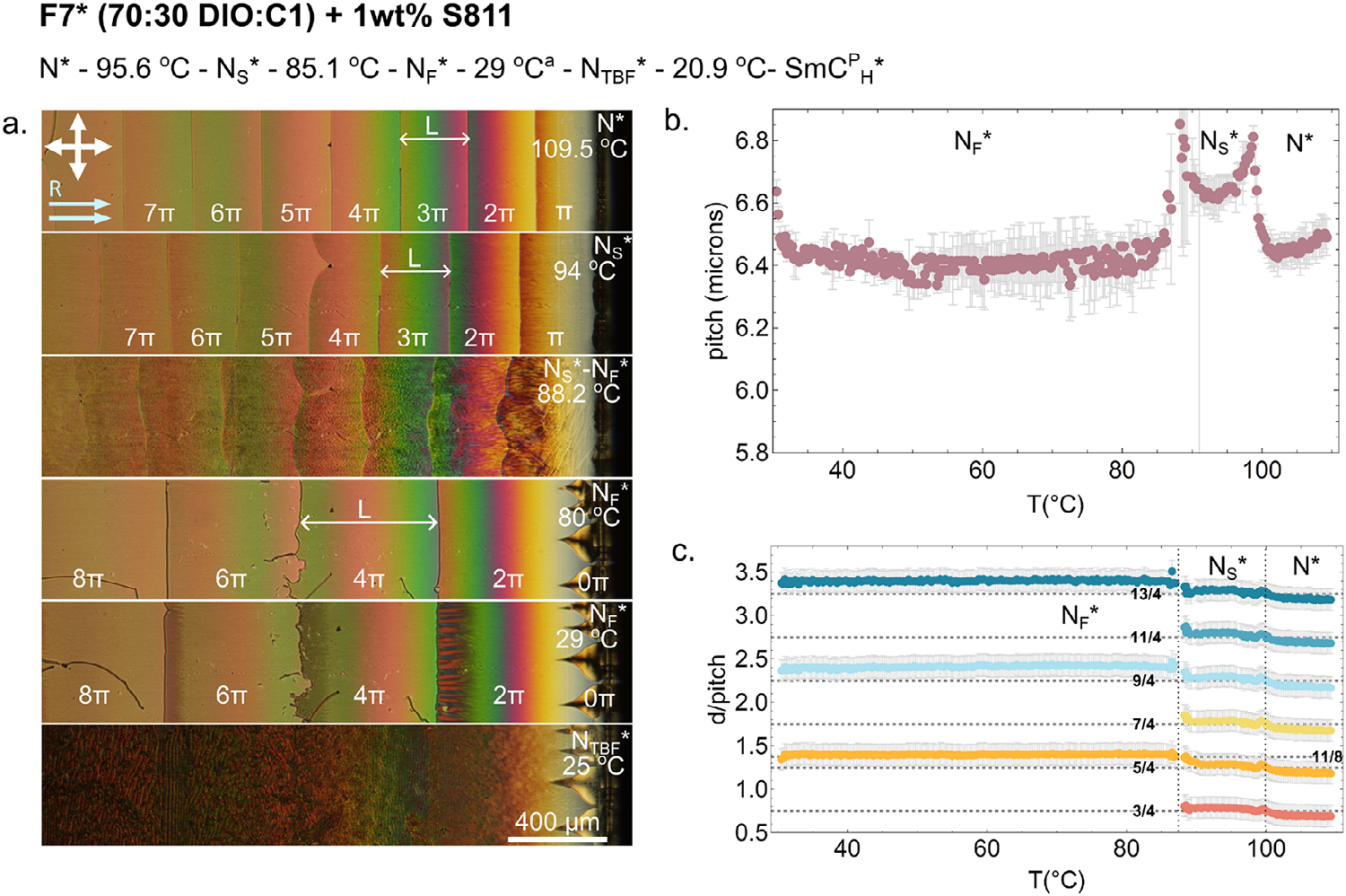
Characterization of F7*. (a) Temperature evolution of Grandjean–Cano texture in a wedge cell (θ = 0.68°) with parallelly rubbed surfaces. The chiral twisted structure adapts to the varying thickness by creating regions of different number of director π‐twist, by jumps of π (N* and N_S_*) and 2π (N_F_*), separated by defect lines. Before the transition to the N_TBF_* phase, evident pretransitional modulated instabilities occur in the hypotwisted part of the GC regions and at the transition, a macroscopic modulated structure develops in the N_TBF_* with period increasing with the cell thickness. (b) Mesophase pitch vs. temperature as determined considering *p* = 2*L*tanθ for the N* and N_S_* phases, and *p* = *L*tanθ for the N_F_* phase. (c) Temperature dependence of the position of the GC defect lines in terms of cell thickness (*d*) vs. pitch.

We additionally evaluated the thicknesses (*d*) at which edge dislocations appear relative to the measured *p* (Figure 2c). While in the N* and N_S_* phases GC lines are located as expected at the approximate thicknesses (2n+1)*p*/4 with a slight increase at the transition between both phases, in the N_F_* they slightly shift to larger thicknesses, approximately by *p*/8. This indicates that in the N_F_* phase, the local structure stretches up to an apparent pitch of 8.8 µm (i.e., under‐twisted or hypotwisted structure) before the 2π–4π dislocation line, and then contracts to half that value across it (i.e., over‐twisted or hypertwisted structure). Such differentiated twisting conditions allow for the observation of a striking N_F_* to N_TBF_* pretransitional behavior (Figure 2a and Movie S2), that is, nucleating from the edge dislocation toward the thinner part, modulated instabilities appear, either 1D at a slight angle or parallel to the 2π–4π and 6π–8π GC line, or 2D as in the 4π–6π one. On entering the N_TBF_* phase, the texture initially gets grainy and subsequently stabilizes into a modulated texture, with an underlying rope‐like texture and overall modulation increasing with the thickness of the confinement (Figures S6 and S7). This macroscopic periodic structure, is equivalent to that reported by Nishikawa et al. [39]. in an N_TBF_ system, in which the chirality is extrinsically imposed by antiparallel boundary conditions. Same observation is shown by Karcz [37]. However, here, we will focus on pretransitional wave‐like distortions observed in the intrinsically chiral N_F_* phase.

### 2.2 Modulated Instabilities in Thin Confinements

We exploit the possibility to control the direction of polarization by the surface rubbing direction [44, 45], to select odd or even numbers of π‐twists through antiparallel or parallel rubbed cells, and by tuning the cell thickness, to achieve hypotwisted structures that promote the observed pretransitional modulated instabilities. In the case of untwisted states, either in the very thin regions of the wedge cell (Figure 2a), or when material is confined in a 2.2 µm cell (*d/p *= 0.32) with parallel rubbing, no pretransitional modulation is observed (Figure S8). We thus turn to the next simplest twisted structure. We studied a hypotwisted 1‐π structure with F7* confined in a 5.4 µm cell (*d/p *= 0.83, where we take *p* = 6.5 µm) with antiparallel rubbing (Figure S9). On slow cooling (0.5°C/min), a few degrees above the N_F_*–N_TBF_* transition, a 1D modulated instability appears with a periodicity of approximately 6 µm and modulation perpendicular to the rubbing direction (Figures 3a and S10 and S11, Movie S3). On cooling, the modulation slowly evolves, that is, the periodicity reduces, characterized by the propagation of dislocations, reaching a minimum value of around 2 µm. This results in the periodic modulation of the effective nonlinear optical coefficient *d*_eff_, as seen in SHG‐M images (Figure 3c and Movie S4). The transition to the lower temperature phase is then marked by the appearance of an additional modulated structure, this time parallel to the rubbing direction of a periodicity around 3 µm, that progressively evolves into a structure (Figure 3a,b) with periodicity equal to the cell thickness and at a slight angle with respect to surface alignment. These textural features in the N*_TBF_ are fully equivalent to those observed for the non‐chiral N_TBF_ under the same antiparallel surface conditions (Figure S5) and correspond to those reported by Nishikawa et al. [39]. On heating, the same behavior is recovered in the N_F_*, although at the onset of the instabilities, the periodicity appears lower than on cooling and monotonically increases with temperature before fading out (Figures 3b and S12). This small hysteresis in the modulation period likely reflects the way the instability is reached on cooling versus heating. As it will be discussed below, on cooling, it develops as the bend elastic constant decreases *K*_3_ on approaching the N*_TBF_ phase, whereas on heating it emerges as *K*_3_ it increases following the transition back to the N_F_*. As a result, elastic stresses are accommodated differently under the same confinement and surface anchoring conditions, and the wavelength continuously adapts to the evolving elastic constants. Finally, this behavior can be compared with the hypertwisted 2‐π structure obtained when F7* is confined in a 5.1 µm cell with parallel rubbed surfaces (Figure S13). In this case, no pretransitional instabilities are observed, evidencing that the stretched twisted structure (Figure 3a–c) is required for the instabilities to appear. On entering the N_TBF_* a periodic texture compatible with the hierarchical 3D twist structures proposed by Nishikawa et al. for the non‐chiral system is observed [39].

**FIGURE 3:**
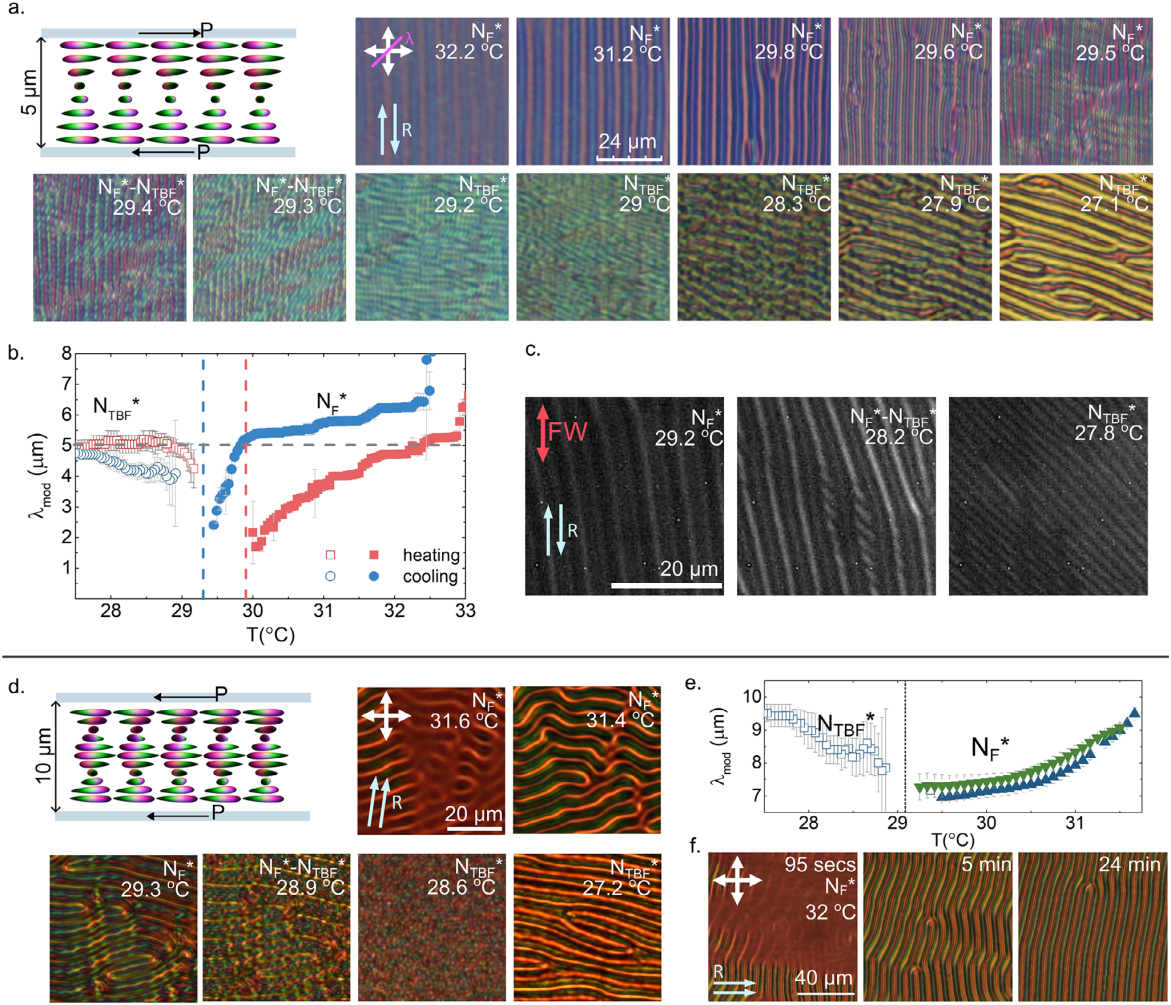
Elastic instabilities in hypo‐twisted π and 2π‐twist structures. (a) Sketch of the π‐twist equilibrium structure in a 5.4 µm antiparallel rubbed cell in the N_F_* phase and sequence of POM images on cooling, from the onset of the instabilities in the N_F_* phase down to the N_TBF_* phase. Double‐headed white arrows indicate the direction of polarizers, and light‐blue arrows indicate direction of surface rubbing (R). (b) Temperature evolution of the modulation of the instabilities (N_F_*) and of the periodic macrostructure (N_TBF_*) in both heating and cooling runs. Modulation was calculated from the dominant peak in the Fourier transform of intensity profiles, as described in the Experimental Section and Figure S11. (c) SHG microscopy images of the instabilities in the N_F_* and at the transition to the N_TBF_*. Full sequence in Movie S3. The red double‐headed arrow indicates direction of polarization of the fundamental beam. (d) Sketch of the 2π‐twist equilibrium structure in a 10.1 µm parallel‐rubbed cell in the N_F_* phase and sequence of POM images on cooling, from the onset of the instabilities in the N_F_* phase down to the N_TBF_* phase. (e) Temperature evolution of the modulation of the instabilities (N_F_*) and of the periodic macrostructure (N_TBF_*) on cooling. Both sets of triangles correspond to areas in which modulation appears tilted in complementary directions with respect to the rubbing direction. (f) Temporal stabilization of instabilities parallel to the rubbing direction at the onset temperature.

The same analysis was conducted for a hypotwisted 2‐π structure using a 10.1 µm cell (*d/p *= 1.55) with both surfaces rubbed in the same direction (Figure 3d–f). Given the confining ratio, on cooling from the N* and N_S_* phases, the LC cell is predominantly covered by 4‐π structure, with small islands of 2‐π structure. These islands originate during the N_S_*–N_F_* transition, when the system evolves from a 3‐π equilibrium structure to even‐π configurations imposed by surfaces, and become locally pinned at defects or surface imperfections, remaining stable with temperature within the predominantly 4‐π background (Figure S14). On approaching the N_F_* to N_TBF_* transition, modulated instabilities appear in such islands, while the surrounding texture remains unaltered, allowing for a clear visualization of the importance of the elastic field in the formation of the instabilities. In this temperature interval, 2‐π regions expand before the transition, but drastically in the N_TBF_* phase, indicating an increase of the pitch associated with the emergence of heliconical order. Subsequent thermal treatment cooling and heating across the transition allows stabilization of the 2‐π ground structure across the entire cell (Figure S15). On slow cooling conditions (0.5°C/min), as the system evolves toward the transition, the modulation grows in two directions, at a slight angle from the rubbing direction, that is, mostly perpendicularly with respect to the previous 1‐π twist case. In contrast, when the sample is cooled to the onset of the instabilities and the temperature is subsequently stabilized for a longer period (Figure 3f) the modulation evolves into a much more oriented 1D morphology with a single preferred orientation.

The interplay between surface anchoring and modulation directions can be further explored in 1/2‐π twisted structures (Figure 4a–c), achieved in left‐handed 5 µm cells (*d/p *= 0.77) with buffing directions at 90° (Figure S16). Under these conditions, the undulations emerge at 45°, with a decreasing period on cooling.

**FIGURE 4:**
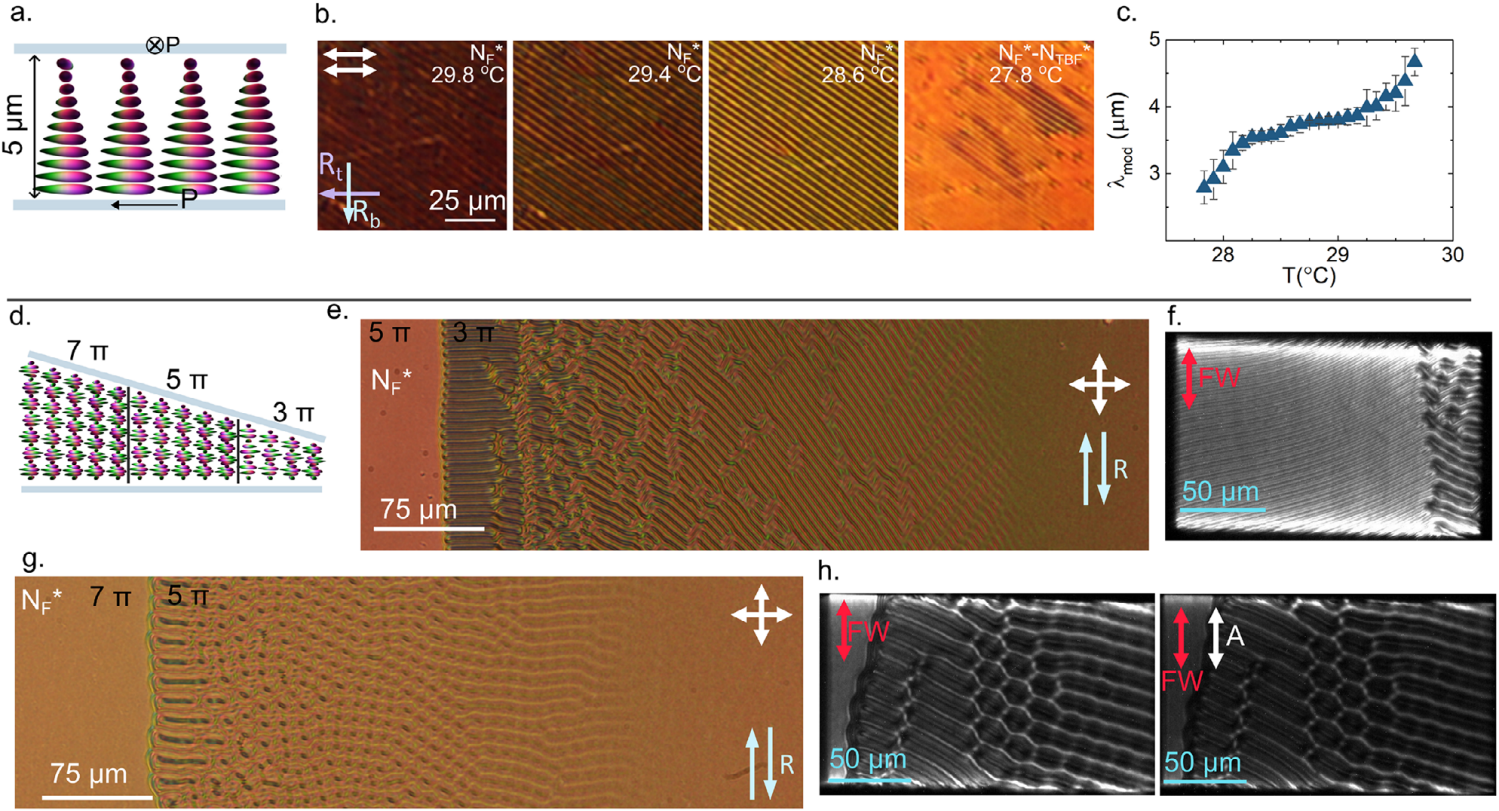
Elastic instabilities in hypo‐twisted π/2, 3π‐twist, and 5π‐twist structures. (a) Sketch of the π/2‐twist equilibrium structure in a 5 µm 90 left‐handed rubbed cell in the N_F_* phase. (b) Sequence of POM images on cooling, from the onset of the instabilities in the N_F_* phase down to the N_TBF_* phase. Double‐headed white arrows indicate the direction of polarizers, and light‐blue arrows indicate the direction of surface rubbing (R). (c) Temperature evolution of the observed modulation in the N_F_*. (d) Sketch of the custom‐made wedge cell (θ = 0.22°) with antiparallel rubbing perpendicularly to the wedge direction. (e) POM texture in the N_F_* phase at 29.7°C in the boundary between the 5π‐ and 3π‐twisted structures. (f) Second Harmonic Generation (SHG) microscopy image in the 3π‐twisted GC region, in an equivalent area to that in (e). The red double‐headed arrow represents the polarization of the incoming fundamental wave. (g) POM texture in the N_F_* phase at 29.7°C in the boundary between the 7π‐ and 5π‐twisted structures, showing a hierarchy of 1D and 2D instabilities. (h) SHG microscopy images in the vicinity of the 7π–5π GC line, reflecting the spatial structuring of the effective second‐order nonlinear susceptibility. The red double‐headed arrow represents the polarization of the incoming fundamental wave, while the white arrow (A) represents the direction of the analyzer for the right panel.

### 2.3 Modulated Instabilities in Thicker Confinements

To investigate pretransitional undulations in higher twisted structures, we fabricated a homemade wedge cell with a smaller wedge angle (θ  =  0.22°) and antiparallel rubbing perpendicular to the wedge direction. This anchoring geometry imposes opposite orientations of ***P*** at the surfaces, with GC lines now separating regions with 3π, 5π, and 7π (overview and Zoom‐ins in Figures S17, S18, and S20). The temperature was then held 0.5°C below the instability onset, allowing the patterns to stabilize.

Along the GC 3π–5π defect line (Figure 4e,f), a 1D periodic modulation appears, with stripe orientation varying with the local thickness. Near the GC‐line, the modulation is perpendicular to the surface alignment, whereas in the thinner part of the 3π twisted region, the stripes rotate to about −45°. At the same time, the periodicity of the modulation slightly reduces from approximately 6.6 to 5 µm, that is, from values ∼p to ∼0.8p. It should be noted that other regions of the cell display additional periodic morphologies with increased complexity. In the thicker region, around the GC 5π–7π defect line (Figure 4g,h), different morphologies can be observed, from 1D modulations by the GC line, to 2D square and 2D hexagonal patterns with periodicities ranging between 8 and 10 µm depending on the local thickness. All these structures are clearly resolved in SHG‐M images, which show a pronounced spatial modulation of the SHG signal that closely follows the periodic morphology of the undulation patterns, with the overall signal being maximized when the fundamental wave polarization is parallel to the surface alignment direction (Figures S18 and S20). Since the spontaneous polarization can be assumed to be ***P***  = *P*_0_ ***n*** in the N_F_* phase, the periodic modulation of the director field together with the modulation of the SHG intensity, is consistent with a periodic variation of the local polarization direction. This shows that the elastic instability produces a spatially modulated polarization field, driven by the director deformation. As will be discussed below, the mechanism involved provides a new route for achieving self‐organized periodic nonlinear optical media.

### 2.4 Modelling of Instabilities

The above described observations differ significantly from recently reported phenomena in related systems. Particularly, Nishikawa et al. stabilized the heliconical polar structure of the heliconical HEC mesophase (N_TBF_) in a non‐chiral system between antiparallelly rubbed substrates, imposing an extrinsically twisted configuration [39]. They observed that the coexistence of extrinsic (twisted structure imposed by the boundary conditions) and intrinsic (heliconical) twists leads to a triple chiral state, observed in POM as a striped texture, with stripes oriented perpendicularly to the rubbing direction. This structure occurs in the low temperature N_TBF_ (HEC) phase, similarly to the observations here down in the N_TBF_*, with the difference that in the present case, the helical twist is also intrinsic. In our chiral system, although we see compatible behavior in the N_TBF_* phase, we focused on the instabilities that appear in the higher‐temperature N_F_* phase. These are not observed in the non‐chiral N_F_ system, in which the twist is imposed by boundary conditions and does not correspond to a bulk energetic minimum and the material does not need to adapt an intrinsic pitch, so no bulk energetic penalty arises with thickness beyond the cost of accommodating the imposed boundary twist. In the N_F_* system, in contrast, the twist is energetically selected in the bulk and competes with confinement.

In a more similar system, Zhou et al. [46] reported the formation of a 2D grid‐like pattern in the N*_F_ phase of chiral systems based on RM734 and homologues. These systems are similar to ours in that they are intrinsically chiral, but differ in that they are not reported to undergo a transition to the heliconical N_TBF_* phase and instead crystallize. However, the authors identified a critical thickness (dependent on the helical pitch) above which these structures appear and modelled their emergence using a free energy functional including elastic, compression and polar contributions. Their model returns a critical pitch (2π/*q*_c_) and the condition of a critical thickness *d*_c_ =  2πN/*q*_c_ which prevents the formation of the modulated structure in samples thinner than the material helical pitch. Strikingly, in the case of our observations, although a 2D‐grid‐like pattern is observed in the thicker parts of the sample, the morphology is different from that reported by Zhou et al. [46] and 1D modulations are reproducibly obtained in thin confinements, even in thicknesses comparable to or smaller than the helical pitch, always in hypotwisted confinement. The observed behavior in our case is similar to that reported for the nonpolar nematic phase in twist‐bend nematic dimers, which was successfully explained in terms of elastic energy arguments [24, 26]. For the parent non‐chiral F7 material considered here, we recently reported a marked softening of the bend elastic constant *K*_3_ on approaching the N_F_ to N_TBF_ phase [47]. While Figure 5a shows the reported temperature dependence of the *K*_3_/*K*_2_ ratio, *K*_2_ is shown to remain practically constant before the transition, with the pronounced reduction thus driven by the softening of *K*_3_.

**FIGURE 5:**
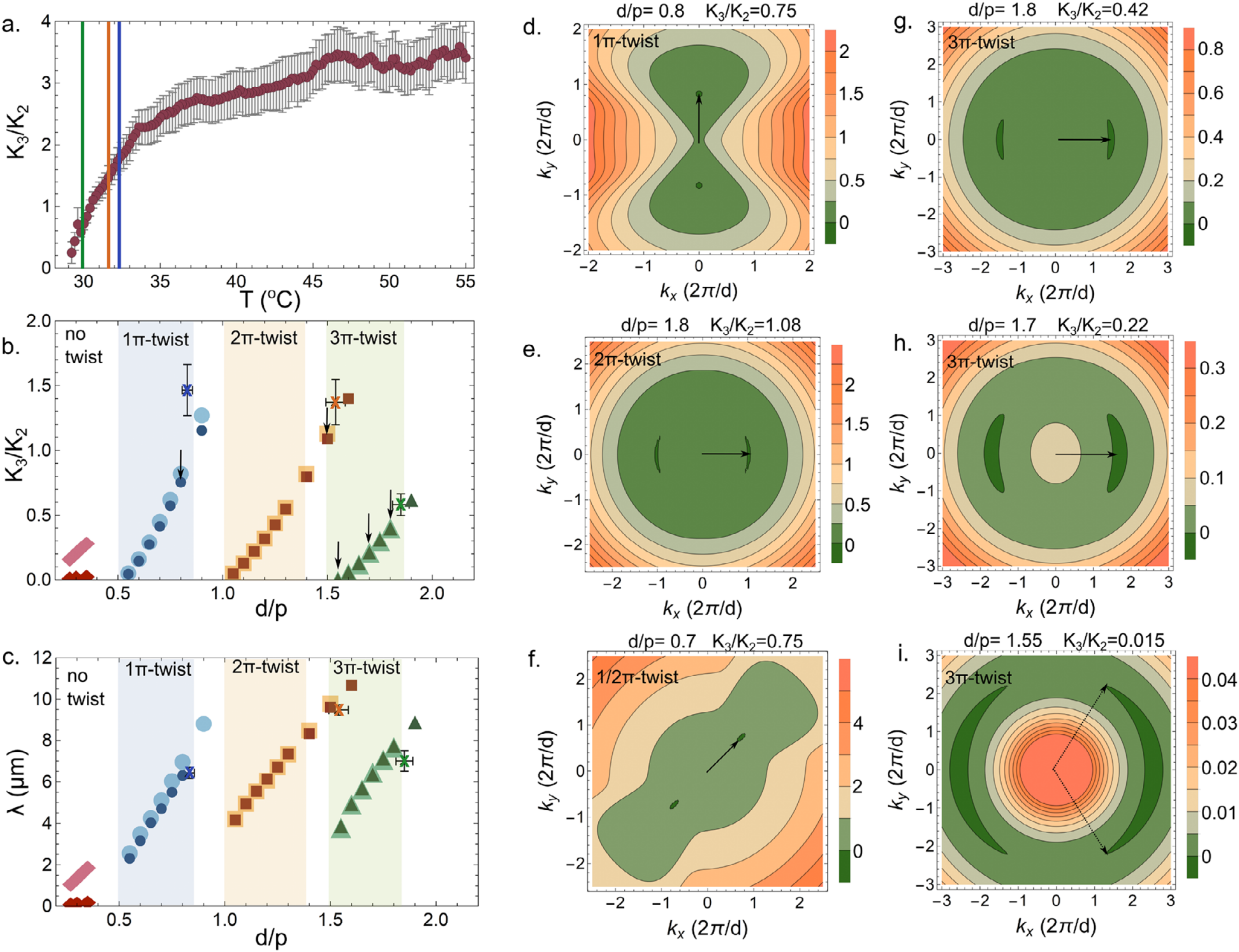
Numerical predictions vs. experimental observations of twist‐induced elastic instabilities. (a) Temperature dependence of the ratio between the bend and twist elastic constants *K*_3_/*K*_2_ as reported in [47], used to compare with the model results. (b) Critical *K*_3n_ *= K*_3_/*K*_2_ as a function of confinement ratio *d*/*p* for 0π‐, 1π‐, 2π‐, and 3π‐ twisted ground structures as determined from linear stability analysis. Lighter symbols correspond to *K*_1n_ = 2.5, while darker ones correspond to *K*_1n_ = 100. Crosses mark for the experimental *d/p* investigations, the corresponding *K*_3n_ value as obtained from (a). Vertical arrows mark the instability points for the corresponding parameter sets used for the calculations shown in panels (d–i). (c) Calculated instabilities modulation for the 0π‐, 1π‐, 2π‐, and 3π‐ twisted ground structures are compared with the experimentally observed ones at the onset of instabilities for the different confinements. (d–i) Two‐dimensional maps of the lowest calculated eigenvalues as a function of the instability wavevector (*k*_x_ *,k*_y_) for different confinement ratios and twisted ground structures. For 1π‐ (d), 2π‐(e), and 1/2π‐(f) structures, the anisotropic minima select stripe‐like modulations with modulation perpendicular, parallel, and at 45° with respect to surface alignment direction (along *x*), respectively. (g–i) For the 3π‐twist structure, the dispersion shows a strong dependency on the confinement ratio, showing a shallow minimum allowing for multiple accessible modulation directions.

Here, we investigate the emergence of instabilities by determining stability diagrams in terms of confinement ratio *d*/*p* and the elastic ratio *K*_3n_ **= *K*_3_/*K*_2_. The director field is parametrized in terms of two angular variables, θ(***r***) in the *x*–*y* plane and α(***r***) in the out of plane direction, ***n***  =  (cos α(***r***)cos θ(***r***), cos α(***r***)sin θ(***r***), sin α(***r***)). The analysis is restricted to defect‐free bulk textures, where the polarization is assumed to be ***P***  = *P*_0_ ***n***, with a constant magnitude of *P*_0_. The base state depends only on the coordinate *z* along the cell thickness, and its stability is analyzed with respect to small periodic perturbations of the type $\alpha \left(r\right)=\alpha_{0}\;\left(z\right)+\delta \alpha \left(z\right)e^{iq\cdot r_{⊥}}+c.c.$ and $\theta \;\left(r\right)=\theta_{0}\;\left(z\right)+\delta \theta \left(z\right)e^{iq\cdot r_{⊥}}+c.c.$, with the wavevector ***q***  =  (*k*_x_,*k*_y_) and ***r***_⊥_ =  (*x*, *y*). To account for the different N‐twisted structures, we take θ_0_ (*z*) =  Nπ*z*/*d* and α_0_ (*z*) =  0.

We first establish the equilibrium configuration by numerical minimization of the Frank–Oseen free energy

(1) $$\begin{array}{ccc} & & F=\int \left(\frac{1}{2}K_{1}{\left(\nabla \cdot n\right)}^{2}+\frac{1}{2}K_{2}{\left(n\cdot \left(\nabla \times n\right)+q_{0}\right)}^{2}\right.\\ & & \left.+\frac{1}{2}K_{3}{\left(n\times \left(\nabla \times n\right)\right)}^{2}\right)\;\mathrm{dV}-\int \frac{1}{2}W{\left(n\cdot n_{s}\right)}^{2}\mathrm{dS} \end{array}$$

where *K*_i_ are the elastic constants, *q*_0_ =  2π/*p* is the intrinsic helical wave number, ***n***_s_ is the preferred direction of ***n*** at the surface of the cell (taken parallel to the substrates), and *W* is the anchoring strength. Consistent with our experimental investigations, strong anchoring is assumed, with *W*  =  1 · 10^−5^ Jm^−2^. Under these conditions, the saddle–splay contribution can be rewritten into a form of surface term, associated with gradients of the director tangent to the substrates [48]. In the present conditions, it then vanishes or remains negligible. This Frank–Oseen continuum description is applied under the assumption of smoothly varying director fields, away from defects and domain walls. The polarization is assumed to be ***P***  = *P*_0_ ***n*** and the electrostatic interactions of bound charges can be accounted for through an effective renormalization of the splay elastic constant [49, 50, 51]. While this represents a simplified description of the polarization field, it is sufficient for our purpose of characterizing the onset of the instability. This approximation has been successfully employed to describe ferroelectric nematic textures, including polar conic structures [51]. To assess the influence of this electrostatic contribution, calculations were performed for *K*_1n_ *= K*_1_/*K*_2_ **= 100 and *K*_1n_ **= 2.5, with the larger value reflecting the electrostatic cost associated with the bound charge density, *ρ*_*b*_ =   − *P*_0_∇ · ***n***, generated by splay deformations in the polar system.

The stability analysis is performed numerically by calculating for each pair of parameters *d/p* and *K*_3_/*K*_2_, the lowest nontrivial eigenvalue and corresponding wavevector ***q***. The onset of the instability is then identified when the minimum eigenvalue becomes negative at a finite ***q***, marking the transition of the undeformed state toward the modulated instability. Stability diagrams for the structures θ_0_ (*z*) =  Nπ*z*/*d* with *N* = 0, 1, 2, and 3 are shown in Figure 5b within the hypotwisted *d/p* bands. For *N* = 0, it is found that *K*_1n_ **= 100 suppresses the instability for the untwisted state, in agreement with observations here. In contrast, reducing the normalized splay elastic constant to *K*_1n_ **= 2.5 restores the instability, indicating that the model predicts its onset when the electrostatic penalty for splay deformations is sufficiently weak, as reported for nonpolar twist‐bend dimers [26]. For the twisted structures, the stability diagrams obtained for *K*_1n_ *= K*_1_/*K*_2_ **= 100 and *K*_1n_ **= 2.5 are nearly identical (Figure 5b), indicating that the instability threshold is largely insensitive to electrostatic stiffening. The model reveals an inverse relationship between the required *K*_3n_ and the deviation of the imposed twist from the intrinsic cholesteric pitch *p*. As the frustration of the natural pitch increases, instabilities are triggered at larger bend elasticity. No unstable solutions are found for the hypertwisted structures (i.e., *d/p *< 0.5, 1, and 1.5). These results are compared with experimental observations for onset conditions and modulation periodicities at *d/p* = 0.8, 1.55, and 1.8, where the corresponding *K*_3n_ values are extracted from Figure 5a at the observed onset temperature. Experimental values are indicated by crosses in Figure 5b,c, and show a very good correspondence with the numerical predictions.

Further insight into the nature of the emerging patterns is obtained by analyzing the spectral landscape in the (*k*_x_ *,k*_y_) plane. Representative plots for twisted states corresponding to the structures with 1π‐, 2π‐, 1/2π‐, and 3π‐ twist across the cell are shown in Figure 5d–i. In the first three cases, two symmetric narrow minima appear, indicating that the instability selects a preferred orientation and therefore favors stripe‐like modulations. The orientation depends on the initial twist structure, perpendicular to the surface direction for 1π, parallel in the case of 2π, and at 45° for 1/2π, in line with our experimental observations. In the case of the 2π‐twisted structure, the deviation of the modulation direction observed under slow cooling conditions can be understood as the consequence of the continuous evolution of *K*_3n_ during the experiment, where the more complex initial twisted structure can influence the selection of modulation orientations according to Figure 5e. The calculated wavelengths are shown in Figure 5c. Altogether, the calculated configurations reproduce not only the experimentally observed characteristic orientation of the instabilities, but also experimentally observed modulation periods. To reproduce the experimental behavior in the wedge cell for the 3π‐structure, the same analysis is performed for different confinement ratios *d/p*. Reducing *d*/*p* reveals a progressive increase in the number of accessible modes with different orientations, together with a slight increase in the critical *k* value, evidenced by the shallow minimum in the spectra. This matches experimental observations shown in Figure 4, where instabilities first emerge from the GC line, and expand on cooling toward the thinner regions while the angle varies and the period decreases. The same analysis is extended for the 5π (Figure S21), revealing a nearly isotropic minimum at a finite wavevector, corresponding to modes with different orientations but similar magnitudes. This allows for different combinations of critical modes, including single‐mode stripes, orthogonal mode pairs, forming square lattices, and triplets, that can give rise to hexagonal lattices, consistent with the hierarchy of patterns observed experimentally.

The weak dependence of the stability diagrams for the twisted structures on *K*_1n_ suggests that the bound charges associated with the considered deformations do not generate a significant electrostatic energy penalty at the onset of the instabilities. To understand this behavior, we reconstruct the 3D director configurations associated with the unstable modes and calculate the corresponding bound charge density, assuming ***P***  = *P*_0_ ***n***. The director fields are obtained by taking the critical instability mode as an initial condition for free energy minimization. The resulting configurations are shown in Figure 6. From the calculated director field and considering *P*_0_ =  5 µC cm^−2^ we then determine the spatial distribution of bound charges *ρ*_*b*_ =   − ∇ · ***P***. Considering a typical ion density value *ρ*_0_ =  1 · 10^23^ ions m^−3^ value for the system free charges, the calculated bound charge density is significantly smaller. We can further consider the Debye screening length $\lambda_{D}=\sqrt{\left(\varepsilon \varepsilon_{0}k_{B}T\right)/\left(2\rho_{0}e^{2}\right)}\;$ and the characteristic length associated with bound charges that can be calculated for a periodic distortion $\xi_{b}=\sqrt{\left(\varepsilon \varepsilon_{0}k_{B}T\right)/(P_{0}\left|\nabla \cdot n|e\right)}$. Considering the maximum values |∇ · ***n*** | for the reconstructed 3D director fields (Figures S22 and S23), the coefficient $\beta =\xi_{b}/\lambda_{D}=\sqrt{\left(2\rho_{0}e\right)/(P_{0}\left|\nabla \cdot n|\right)}\;$ can be estimated to be ∼6.5, 8, and 4.5, for the 1π‐, 2π‐, and 3π‐twisted structures, respectively. Values of *β* > 1 indicate that the density of available mobile charges exceeds the characteristic density of bound charges, implying that Debye screening occurs over a shorter distance than the characteristic length associated with polarization charges. Under these conditions, electrostatic interactions are expected to remain weak. To further assess the role of electrostatic effects in the emergence of the observed instabilities, we prepared an ion‐doped F7* sample, using a modified Rhodamine B ionic dye (Figure 7e), increasing the ion density by one order of magnitude (*ρ*_0_ =  1.2 · 10^24^ ions m^−3^). We repeated the experiments for a pi‐twisted structure confined in a 5.1 µm cell with antiparallel rubbing, equivalent to the conditions considered in Figure 3a–c. Despite the significant increase in the concentration of mobile charges, we observe the same instability behavior and identical types of unstable modes, as shown in Figure 7a–d. The persistence of the same instability behavior despite the modified ionic environment further indicates that electrostatic interactions do not play a dominant role in determining the onset of these instabilities. Instead, the results suggest that the observed modulations are primarily governed by elastic contributions, arising from the competition between the energetic cost of deviating from the bulk‐favored twisted configuration and the relatively low penalty associated with bend deformations. While elastic contributions appear to dominate the onset of the instability, polarization effects may still play an important role in determining the subsequent evolution, metastability, and switching behavior of the modulated structures. A comprehensive theoretical analysis of these additional effects is beyond the scope of this experimental work.

**FIGURE 6:**
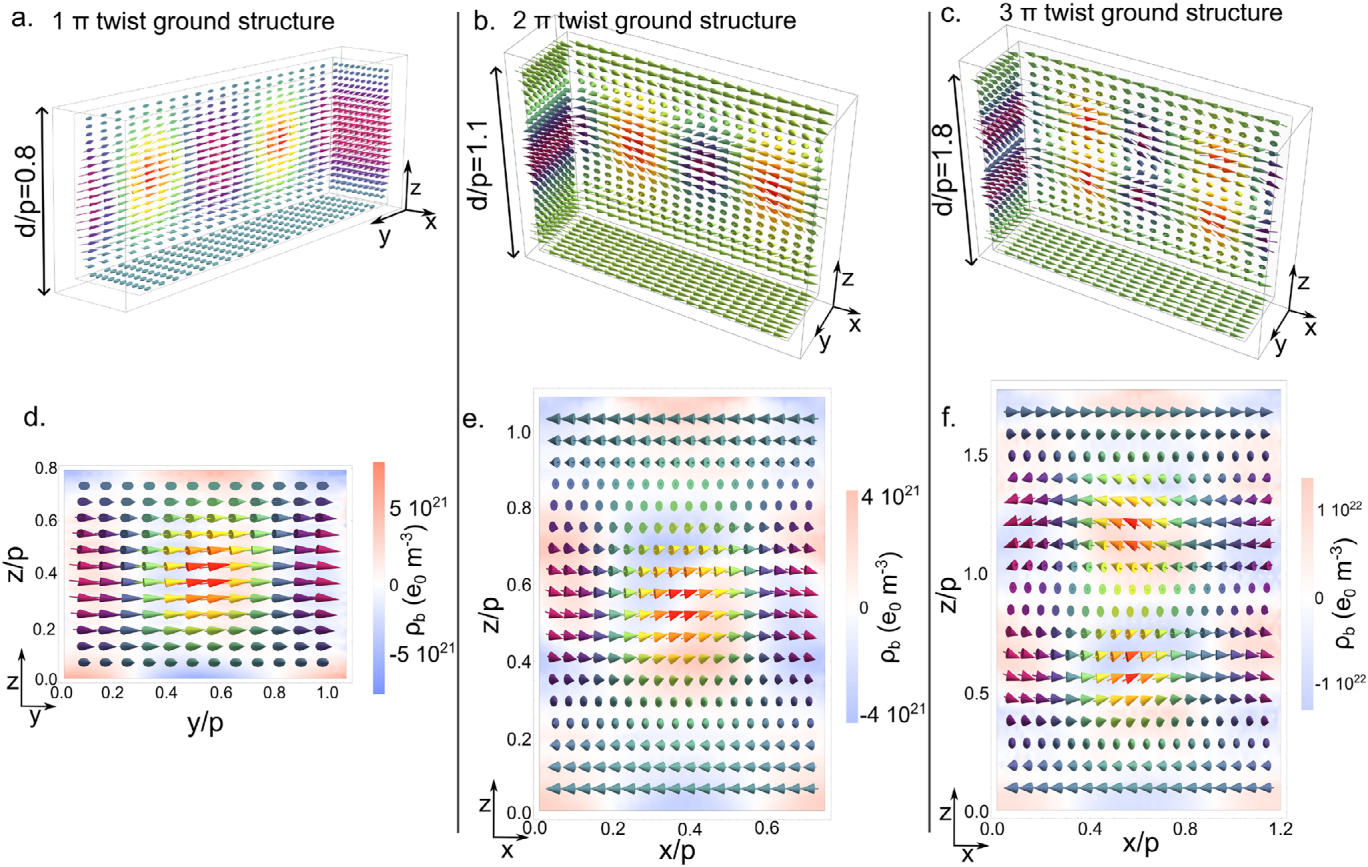
Director fields and bound charge density. (a–c) Director/polarization fields obtained by taking the critical instability mode as an initial condition for free energy minimization for 1π‐, 2π‐, and 3π‐ twist structures, where *x* is along the cell surface alignment direction and *z* is along the cell thickness. The color of the arrows indicates differences in the *z* component of the local director. (d–f) Projected director/polarization field for the structures in (a–c) and bound charge density as calculated from *ρ*_*b*_ = −∇ · ***P*** considering ***P*** = *P*_0_ ***n*** and *P*_0_ = 5 µC/cm^2^.

**FIGURE 7:**
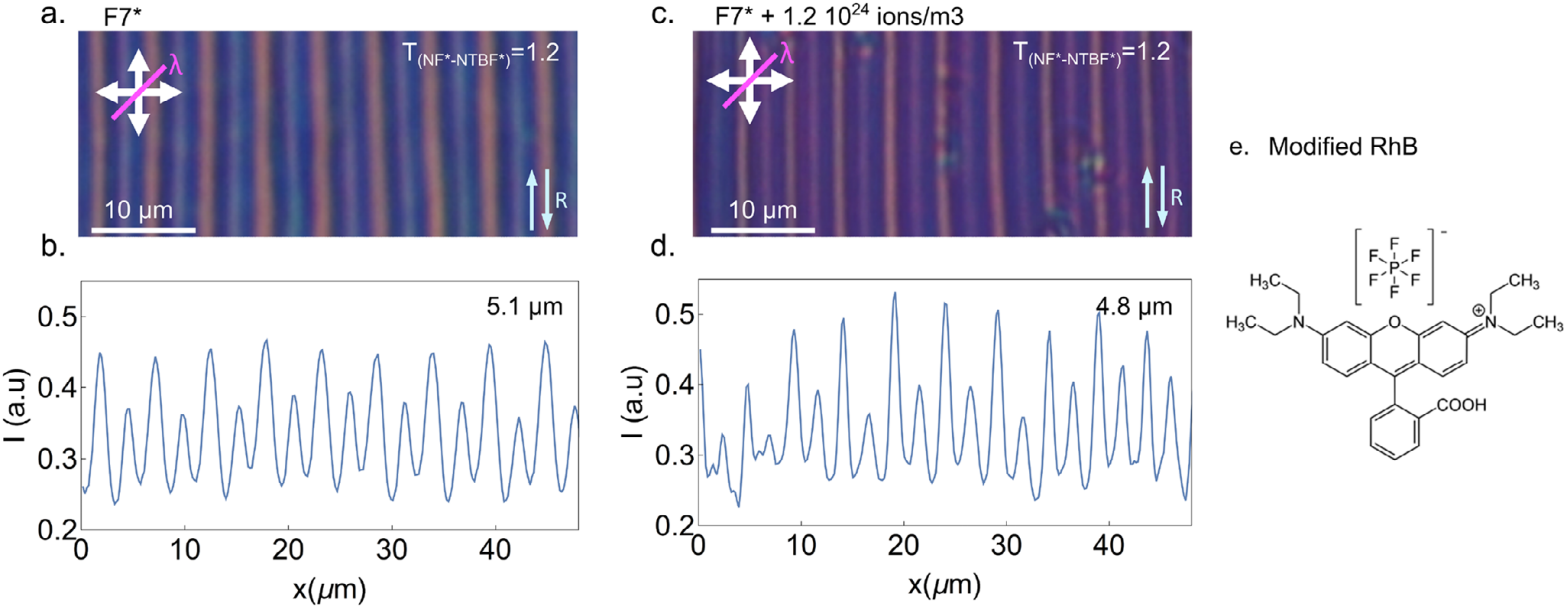
Comparison of instabilites for F7* and ion‐doped F7* in hypo‐twisted π‐twist structures. (a) POM image for F7* confined in a 5.4 µm thick cell with antiparallel rubbing 1.25° above the N_F_*–N_TBF_* transition temperature. (b) One‐dimensional intensity profile as a function of x (horizontal), obtained by averaging the pixel intensity over the entire y (vertical) direction for the image in (a). (b) POM image for ion‐doped F7* in a 5.1 µm thick cell with antiparallel rubbing 1.25° above the N_F_*–N_TBF_* transition temperature. (d) One‐dimensional intensity profile as a function of x (horizontal), obtained by averaging the pixel intensity over the entire y (vertical) direction for the image in (c). (e) Molecular structure of the modified Rhodamine B ion dye used in (c and d). In (a–c), double‐headed white arrows indicate the direction of polarizers, light‐blue arrows indicate direction of surface rubbing (R) and magenta line indicates the slow axis of the inserted λ plate.

## 3 Outlook

The good correspondence between the model and our experimental observations shows that the observed instabilities in F7* can be understood in terms of elastic energy considerations, demonstrating how these instabilities develop under the electrostatic constraints imposed by ferroelectric order. In this respect, this is analogous to what happens in twist‐bend dimers, where the elastic field, effectively played by the elastic frustration and the low energetic cost of bend deformations, directly generates modulated director fields without the need for external electric or magnetic fields [24, 26]. This behavior is enabled by the pronounced softening of the bend elastic constant prior to the transition to the N_TBF_* phase. However, in the present case, the coupling between director distortions and spontaneous polarization implies that elastic instabilities in ferroelectric nematic fluids naturally generate periodic polarization structures, which are expected to carry an electrostatic cost. It is often assumed that such a cost can only be compensated by other electrostatic contributions, since the corresponding elastic energy scale is comparatively small. The present mechanism highlights a regime in which the electrostatic penalty is sufficiently small that elastic effects are responsible for the onset of instabilities. Additional contributions, such as flexoelectric and electrostatic effects, may influence the detailed structure that develops beyond the onset of the instability. This provides a direct route for creating polarization patterns through purely mechanical or geometric control of the director configuration. The temperature stability of the patterns could be improved by selective doping of FNLC materials with bend dimers, as has been proven efficient in the past for other systems [52, 53]. Alternatively, the use of compatible reactive monomers could provide a route toward polymerizing and thereby stabilizing the resulting structures. These approaches, together with surface‐patterned alignment strategies, offer promising avenues for further investigation. This ability to generate polarization‐modulated states through purely elastic and geometric control, translating into spatial structuring of the effective second‐order nonlinear susceptibility, offers a powerful approach toward reconfigurable photonic materials.

More broadly, these results highlight FNLCs as a promising soft‐matter platform for exploring polar topological structures. The coexistence of chirality, polarity, and strong elastic anisotropy allows the emergence of polarization textures conceptually related to those topological formation states (from vortices to hopfions) observed in solid ferroelectrics [54, 55, 56, 57, 58, 59]. However, the soft matter nature enables continuous tuning of these structures through confinement, temperature, and elastic parameters in a more accessible way, offering a powerful tool for probing fundamental aspects of polarization topology in condensed matter.

## 4 Experimental Section

### 4.1 Materials

The synthesis of the liquid‐crystalline material DIO (2,3′,4′,5′‐tetrafluoro‐[1,1′‐biphenyl]‐4‐yl 2,6‐difluoro‐4‐(5‐propyl‐1,3‐dioxan‐2‐yl)benzoate) has been carried out according to the description given in reference [28]. The molecular structure, together with the phase sequence are presented in Figure S1. Description of the synthesis of the liquid‐crystalline material C1 can be found in reference [40]. The molecular structure and the phase sequence are also given in Figure S1.

F7* mixture was prepared by mixing F7 binary mixture (70:30 DIO:C1) and the commercial chiral dopant S811 (Sigma–Aldrich), with the later at 1 wt%. F7 and S811 were weighted separately, adding the required volume of a S811 solution at 1 mg/ml into F7. Additional chloroform was then added for full dissolution, and then the mixture was sonicated for 5 min. After solvent evaporation, the mixture was let to dry for 2 h at 70°C. Due to the 1,3‐dioxane unit in both materials, the samples were always maintained below 120°C to avoid changes in the molecular structure.

The ion‐doped F7* sample was prepared as follows. First, a solution of Modified Rhodamine B (mRhB) dissolved in acetone was prepared at a concentration of 0.51 mg/mL. Then, 34 µL of this solution was added to 20 mg of F7* to achieve the desired mRhB number density of 1.2 · 10^24^ ions m^−3^. The mixture was subsequently heated to approximately 80°C to evaporate the acetone and was left to dry for 1 h to ensure complete solvent removal.

In this work, we employed both commercial and custom‐made liquid crystal cells. Commercial cells were acquired from EHC, with cell gaps ranging from 2 to 25 µm (KSRP D‐type) and combining parallel or antiparallel rubbing (spin‐coated polyimide LX‐1400 from Hitachi–Kasei) as specified in the results section. EHC wedge cells with tanθ  =  0.0115 according to specifications were used, which were subsequently calibrated. The dihedral angle was determined from the interference fringes in the polarizing optical microscope in reflection using an interferometric filter with a central wavelength of 532 nm. The temperature dependence of the wedge cells' slope was calibrated before filling, detecting only a slight change above 85°C, accounting for a max 1.2% change at 110°C, which was restored after cooling. Homemade cells were built with soda‐lime glass 1 × 2 cm coated with indium‐tin‐oxide treated by spin‐coating with a 30% solution of polyimide SUNEVER 5291 (Nissan) film, and rubbed to achieve orientational in‐plane anchoring (planar alignment) of the LC. To build the wedge cell, glass bead spacers of 8 µm in the thin edge and 50 µm Mylar film were employed. Dihedral angle was measured to be 0.22°.

### 4.2 High‐Resolution AC Calorimetry

High‐resolution AC calorimetric measurements were carried out using a home‐built experimental setup. A comprehensive description of the method is provided in [60]. The system offers thermal stability better than 0.1 mK and allows slow cooling and heating scans. The usual AC mode of operation precisely yields the heat capacity dependence on temperature for a second‐order (continuous) phase transition. In the case of a first‐order (discontinuous) transition, the phase coexistence region is indicated by an anomalous behavior of the phase shift between the applied AC power and the temperature oscillations of the sample. The latent heat can be determined by performing additional relaxation runs. A sample with a mass of 30 mg was loaded into a silver cell, to which a heater and a thermistor were attached on each side. Prior to measurements, the sample was heated to the isotropic phase at 110°C. A cooling run was then carried out at a rate of 350 mK/h, through the temperature range where the N–N_S_ and N_S_–N_F_ transition was expected. Subsequently, the sample was cooled at a faster rate down to 42°C, where measurements commenced at a cooling rate of 400 mK/h.

### 4.3 Diffraction Experiments

Structural characterization of the mesophases was performed via x‐ray diffraction experiments. Two‐dimensional diffraction diagrams were recorded using a Stoe Stadivari goniometer equipped with a Genix3D microfocus generator (Xenocs) and a Dectris Pilatus 100 K detector using Cu K_𝛼_ radiation (*𝜆* = 1.5418 Å). A nitrogen‐gas Cryostream controller (Oxford Cryosystems) allowed for a temperature control of about 0.1°C. The materials were introduced at 90°C in glass capillary tubes of 0.6 mm in diameter, which were continuously rotated during pattern acquisition.

To investigate the periodicity of the heliconical N_TBF_ phase, an optical bench equipped with a 448 nm laser pointer was used. The sample was held inside a heating stage for temperature control (Instec HCS302 hotstage with mk2000B temperature controller) over which a hemispherical plastic screen was placed. An aperture was made to allow the direct beam to pass through without scattering. The diffracted beams were observed as spots on the screen, and their angular positions were estimated from angular marks calibrated on the screen surface.

### 4.4 Polarizing Optical Microscopy and Analysis

POM experiments were performed in an Optiphot‐2 POL Nikon microscope. Images and videos were recorded with a Canon EOS M200 camera. The sample was held in a heating stage (Instec TS62) together with a temperature controller (STC200, Instec).

The modulation periodicity of 1D instabilities was determined from polarized optical microscopy images by selecting 20 adjacent regions of interest (140 pixels wide) within a localized area of the image to minimize the effects of temperature gradients. For each region, the image intensity was averaged along the direction perpendicular to the observed modulation to obtain a 1D intensity profile, and the corresponding Fourier spectrum was calculated (Figure S11). The modulation period and its uncertainty were determined from the average position and standard deviation of the dominant spatial‐frequency peak.

### 4.5 Second Harmonic Generation Microscopy

Second Harmonic Generation microscopy (SHG‐M) was performed using a custom‐built sample‐scanning microscope with an Erbium‐doped fiber laser (C‐Fibre A 780, MenloSystems, 785 nm, 95 fs pulses at a 100 MHz repetition rate) as the laser source. Average power is adjusted using an ND filter depending on the experimental needs. A combination of galvo mirrors and a long‐working distance objective (Nikon CFI T Plan SLWD, NA 0.3) is used to scan the focused beam in the sample plane. The scanning frequencies are much higher (a few 100 Hz) than the imaging frame rate (a few Hz). A detailed description of the setup is available in reference [61]. A long‐working‐distance 20× objective (Nikon CFI T Plan SLWD, NA 0.3) collects the light coming from the sample and a set of 700 nm short‐pass and 400 nm band‐pass filters eliminates the fundamental IR light and any possible fluorescence signal. The final SHG‐M images are acquired with a high‐performance CMOS camera (Grasshopper 3, Teledyne Flir) with a typical integration time of 250 ms, varying dimensions in pixels, and a resolution of 0.285 µm/pixel, or in case of fast scanning 1.12 µm/pixel.

## Author Contributions

**Anej Sterle**: writing – review and editing, investigation, and validation. **Peter Medle Rupnik**: investigation, validation, and writing – review and editing. **Luka Cmok**: investigation and writing – review and editing. **Aitor Erkoreka**: investigation and writing – review and editing. **Marta Lavrič**: investigation and writing – review and editing. **Natan Osterman**: methodology and writing – review and editing. **Calum J. Gibb**: investigation and writing – review and editing. **Jordan Hobbs**: writing – review and editing and investigation. **Josu Martinez‐Perdiguero**: investigation and writing – review and editing. **Richard J. Mandle**: investigation and writing – review and editing. **Alenka Mertelj**: formal analysis, writing – review and editing, and funding acquisition. **Nerea Sebastián**: conceptualization, investigation, writing – original draft, formal analysis, visualization, data curation, validation, writing – review and editing, and funding acquisition.

## Conflicts of Interest

The authors declare no conflicts of interest.

## Supporting information




**Supporting File 1**: adma75002‐sup‐0001‐SuppMat.pdf.

**Supporting File 2**: adma75002‐sup‐0002‐MovieS1.mp4.

**Supporting File 3**: adma75002‐sup‐0003‐MovieS2.mp4.

**Supporting File 4**: adma75002‐sup‐0004‐MovieS3.mp4.

**Supporting File 5**: adma75002‐sup‐0005‐MovieS4.mp4

## Data Availability

The data that support the findings of this study are openly available in Zenodo at https://zenodo.org/, reference number 10.5281/zenodo.19396995.

## References

[1] N. C. Seeman and H. F. Sleiman , “DNA Nanotechnology,” Nature Reviews Materials 3 (2017): 17068, 10.1038/natrevmats.2017.68.

[2] Y. Mai and A. Eisenberg , “Self‐Assembly of Block Copolymers,” ChemSocRev 41 (2012): 5969, 10.1039/C2CS35115C. 22776960

[3] A. H. Gröschel and A. H. E. Müller , “Self‐Assembly Concepts for Multicompartment Nanostructures,” Nanoscale 7 (2015): 11841, 10.1039/C5NR02448J. 26123217

[4] C. Tschierske , “Development of Structural Complexity by Liquid‐Crystal Self‐Assembly,” Angewandte Chemie International Edition 52 (2013): 8828, 10.1002/anie.201300872. 23934786

[5] L.‐L. Ma , C.‐Y. Li , J.‐T. Pan , et al., “Self‐Assembled Liquid Crystal Architectures for Soft Matter Photonics,” Light: Science & Applications 11 (2022): 270, 10.1038/s41377-022-00930-5. PMC9470592 36100592

[6] M. Vellaichamy , U. Jagodič , J. Pišljar , et al., “Microscale Generation and Control of Nanosecond Light by Light in a Liquid Crystal,” Nature Photonics 19 (2025): 758, 10.1038/s41566-025-01693-2. PMC12226344 40621151

[7] G. Poy , A. J. Hess , A. J. Seracuse , M. Paul , S. Žumer , and I. I. Smalyukh , “Interaction and Co‐Assembly of Optical and Topological Solitons,” Nature Photonics 16 (2022): 454, 10.1038/s41566-022-01002-1.

[8] C. Meng , J.‐S. Wu , and I. I. Smalyukh , “Topological Steering of Light by Nematic Vortices and Analogy to Cosmic Strings,” Nature Materials 22 (2023): 64, 10.1038/s41563-022-01414-y. 36456872

[9] W. Helfrich , “Deformation of Cholesteric Liquid Crystals with Low Threshold Voltage,” Applied Physics Letters 17 (1970): 531, 10.1063/1.1653297.

[10] J. P. Hurault , “Static Distortions of a Cholesteric Planar Structure Induced by Magnetic or Ac Electric Fields,” Journal of Chemical Physics 59 (1973): 2068, 10.1063/1.1680293.

[11] N. A. Clark and R. B. Meyer , “Strain‐Induced Instability of Monodomain Smectic A and Cholesteric Liquid Crystals,” Applied Physics Letters 22 (1973): 493, 10.1063/1.1654481.

[12] B. I. Senyuk , I. I. Smalyukh , and O. D. Lavrentovich , “Undulations of Lamellar Liquid Crystals in Cells With Finite Surface Anchoring Near and Well Above the Threshold,” Physical Review E 74 (2006): 011712, 10.1103/PhysRevE.74.011712. 16907116

[13] C. Blanc , G. Durey , R. D. Kamien , T. Lopez‐Leon , M. O. Lavrentovich , and L. Tran , “Helfrich‐Hurault Elastic Instabilities Driven by Geometrical Frustration,” Review of Modern Physics 95 (2023): 015004, 10.1103/RevModPhys.95.015004.

[14] M. O. Lavrentovich and L. Tran , “Undulation Instabilities in Cholesteric Liquid Crystals Induced by Anchoring Transitions,” Physical Review Research 2 (2020): 023128, 10.1103/PhysRevResearch.2.023128.

[15] L. Tran , M. O. Lavrentovich , G. Durey , et al., “Change in Stripes for Cholesteric Shells via Anchoring in Moderation,” Physical Review X 7 (2017): 041029, 10.1103/PhysRevX.7.041029.

[16] Z. Zheng , Y. Li , H. K. Bisoyi , L. Wang , T. J. Bunning , and Q. Li , “Three‐Dimensional Control of the Helical Axis of a Chiral Nematic Liquid Crystal by Light,” Nature 531 (2016): 352, 10.1038/nature17141. 26950601

[17] P. G. De Gennes , “Calcul De La Distorsion d'une Structure Cholesterique Par Un Champ Magnetique,” Solid State Communications 88 (1993): 1043, 10.1016/0038-1098(93)90292-U.

[18] R. B. Meyer , “Effects of Electric and Magnetic Fields on the Structure of Cholesteric Liquid Crystals,” Applied Physics Letters 12 (1968): 281, 10.1063/1.1651992.

[19] M. Cestari , S. Diez‐Berart , D. A. Dunmur , et al., “Phase Behavior and Properties of the Liquid‐Crystal Dimer 1′′,7′′‐Bis(4‐Cyanobiphenyl‐4′‐Yl) Heptane: A Twist‐Bend Nematic Liquid Crystal,” Physical Review E 84 (2011): 031704, 10.1103/PhysRevE.84.031704. 22060387

[20] V. Borshch , Y.‐K. Kim , J. Xiang , et al., “Nematic Twist‐Bend Phase With Nanoscale Modulation of Molecular Orientation,” Nature Communications 4 (2013): 2635, 10.1038/ncomms3635. PMC3831290 24189583

[21] D. Chen , J. H. Porada , J. B. Hooper , et al., “Chiral Heliconical Ground State of Nanoscale Pitch in a Nematic Liquid Crystal of Achiral Molecular Dimers,” PNAS 110 (2013): 15931, 10.1073/pnas.1314654110. PMC3791744 24006362

[22] J. Xiang , S. V. Shiyanovskii , C. Imrie , and O. D. Lavrentovich , “Electrooptic Response of Chiral Nematic Liquid Crystals With Oblique Helicoidal Director,” Physical Review Letters 112 (2014): 217801, 10.1103/PhysRevLett.112.217801.

[23] J. Xiang , Y. Li , Q. Li , et al., “Electrically Tunable Selective Reflection of Light From Ultraviolet to Visible and Infrared by Heliconical Cholesterics,” Advanced Materials 27 (2015): 3014, 10.1002/adma.201500340. PMC4683668 25821155

[24] S. M. Salili , R. R. Ribeiro de Almeida , P. K. Challa , S. N. Sprunt , J. T. Gleeson , and A. Jákli , “Spontaneously Modulated Chiral Nematic Structures of Flexible Bent‐Core Liquid Crystal Dimers,” Liquid Crystals 44 (2017): 160, 10.1080/02678292.2016.1225836.

[25] R. Walker , D. Pociecha , J. M. D. Storey , E. Gorecka , and C. T. Imrie , “The Chiral Twist‐Bend Nematic Phase (N*TB),” Chemistry—A European Journal 25 (2019): 13329, 10.1002/chem.201903014. 31322779

[26] P. Oswald , “Surface‐Field‐Induced Heliconical Instability in the Cholesteric Phase of a Mixture of a Flexible Dimer (CB7CB) and a Rodlike Molecule (8CB),” Physical Review E 105 (2022): 024704, 10.1103/PhysRevE.105.024704. 35291181

[27] P. Oswald and A. Dequidt , “Heliconical‐Fluctuation‐Induced Compensation Point in the Diluted Cholesteric Phase of Mixtures Containing the Flexible Dimer CB7CB,” Physical Review E 105 (2022): 024707, 10.1103/PhysRevE.105.024707. 35291088

[28] H. Nishikawa , K. Shiroshita , H. Higuchi , et al., “A Fluid Liquid‐Crystal Material With Highly Polar Order,” Advanced Materials 29 (2017): 1702354, 10.1002/adma.201702354. 29023971

[29] R. J. Mandle , S. J. Cowling , and J. W. Goodby , “Rational Design of Rod‐Like Liquid Crystals Exhibiting Two Nematic Phases,” Chemistry—A European Journal 23 (2017): 14554, 10.1002/chem.201702742. PMC5656819 28850751

[30] N. Sebastián , L. Cmok , R. J. Mandle , et al., “Ferroelectric‐Ferroelastic Phase Transition in a Nematic Liquid Crystal,” Physical Review Letters 124 (2020): 037801, 10.1103/PhysRevLett.124.037801. 32031856

[31] X. Chen , E. Korblova , D. Dong , et al., “First‐Principles Experimental Demonstration of Ferroelectricity in a Thermotropic Nematic Liquid Crystal: Polar Domains and Striking Electro‐Optics,” Proceedings National Academy of Science USA 117, no. 25 (2020): 14021–14031, 10.1073/pnas.2002290117. PMC7322023 32522878

[32] H. Nishikawa and F. Araoka , “A New Class of Chiral Nematic Phase With Helical Polar Order (Adv. Mater. 35/2021),” Advanced Materials 33 (2021): 2170270, 10.1002/adma.202170270. 34278630

[33] X. Zhao , J. Zhou , J. Li , et al., “Spontaneous Helielectric Nematic Liquid Crystals: Electric Analog to Helimagnets,” Proceedings National Academy of Science USA 118 (2021): e2111101118, 10.1073/pnas.2111101118. PMC8545456 34642251

[34] C. Feng , R. Saha , E. Korblova , D. Walba , S. N. Sprunt , and A. Jákli , “Electrically Tunable Reflection Color of Chiral Ferroelectric Nematic Liquid Crystals,” Advanced Optical Materials 9 (2021): 2101230, 10.1002/adom.202101230.

[35] C. L. Folcia , J. Ortega , R. Vidal , T. Sierra , and J. Etxebarria , “The Ferroelectric Nematic Phase: An Optimum Liquid Crystal Candidate for Nonlinear Optics,” Liquid Crystals 49 (2022): 899, 10.1080/02678292.2022.2056927.

[36] M. Lovšin , L. Cmok , C. J. Gibb , et al., “Ferroelectric Fluids for Nonlinear Photonics: Evaluation of Temperature Dependence of Second‐Order Susceptibilities,” Advanced Optical Materials 14 (2026): e03018, 10.1002/adom.202503018.

[37] K. Jakub , J. Herman , N. Rychłowicz , et al., “Spontaneous Chiral Symmetry Breaking in Polar Fluid–Heliconical Ferroelectric Nematic Phase,” Science 384 (2024): 1096, 10.1126/science.adn6812. 38843325

[38] H. Nishikawa , D. Okada , D. Kwaria , et al., “Emergent Ferroelectric Nematic and Heliconical Ferroelectric Nematic States in an Achiral “Straight” Polar Rod Mesogen,” Advanced Science 11 (2024): 2405718, 10.1002/advs.202405718. PMC11633337 39099380

[39] H. Nishikawa , D. Kwaria , A. Nihonyanagi , and F. Araoka , “3D Hierarchical Twists in Polar Fluids: Chirality Regulation by Ultralow Electric Field,” Advanced Materials 38 (2026): 13451, 10.1002/adma.202513451. 41128176

[40] C. J. Gibb , J. Hobbs , D. I. Nikolova , et al., “Spontaneous Symmetry Breaking in Polar Fluids,” Nature Communications 15 (2024): 5845, 10.1038/s41467-024-50230-2. PMC11239904 38992039

[41] J. Thoen , G. Cordoyiannis , W. Jiang , G. H. Mehl , and C. Glorieux , “Phase Transitions Study of the Liquid Crystal DIO With a Ferroelectric Nematic, a Nematic, and an Intermediate Phase and of Mixtures With the Ferroelectric Nematic Compound RM734 by Adiabatic Scanning Calorimetry,” Physical Review E 107 (2023): 014701, 10.1103/PhysRevE.107.014701. 36797863

[42] I. I. Smalyukh and O. D. Lavrentovich , “Three‐Dimensional Director Structures of Defects in Grandjean‐Cano Wedges of Cholesteric Liquid Crystals Studied by Fluorescence Confocal Polarizing Microscopy,” Physical Review E 66 (2002): 051703, 10.1103/PhysRevE.66.051703. 12513499

[43] K. Thapa , O. S. Iadlovska , B. Basnet , et al., “Confinement and Magnetic‐Field Effect on Chiral Ferroelectric Nematic Liquid Crystals in Grandjean‐Cano Wedge Cells,” Physical Review E 109 (2024): 054702, 10.1103/PhysRevE.109.054702. 38907387

[44] X. Chen , E. Korblova , M. A. Glaser , J. E. Maclennan , D. M. Walba , and N. A. Clark , “Polar in‐Plane Surface Orientation of a Ferroelectric Nematic Liquid Crystal: Polar Monodomains and Twisted State Electro‐Optics,” PNAS 118 (2021): 2104092118. 10.1073/pnas.2104092118 PMC8179187 34050028

[45] Y.‐T. Hsiao , I. Nys , and K. Neyts , “Lateral Electric Field Switching in Thin Ferroelectric Nematic Liquid Crystal Cells,” Soft Matter 19 (2023): 8617, 10.1039/D3SM00997A. 37916445

[46] J. Zhou , Y. Zou , J. Li , M. Huang , and S. Aya , “Spontaneous Periodic Polarization Wave in Helielectric Fluids,” PNAS Nexus 2 (2023): pgad265, 10.1093/pnasnexus/pgad265. PMC10443924 37614674

[47] A. Erkoreka , J. Martinez‐Perdiguero , L. Cmok , et al., “Flexoelectricity‐Driven Softening of Bend Elasticity Leads to Spontaneous Chiral Symmetry Breaking in a Polar Fluid,” arxiv: 2602.15687 (2026), 10.48550/arXiv.2602.15687.

[48] Ž. Kos and M. Ravnik , “Relevance of Saddle‐Splay Elasticity in Complex Nematic Geometries,” Soft Matter 12 (2016): 1313–1323, 10.1039/c5sm02417j. 26610395

[49] K. Okano , “Electroslatie Contribution to the Distortion Free Energy Density of Ferroelectric Liquid Crystals,” Japan Society of Applied Physics 25 (1986): L846, 10.1143/JJAP.25.L846.

[50] J.‐B. Lee , R. A. Pelcovits , and R. B. Meyer , “Role of Electrostatics in the Texture of Islands in Free‐Standing Ferroelectric Liquid Crystal Films,” Physical Review E 75 (2007): 051701, 10.1103/PhysRevE.75.051701. 17677078

[51] P. Kumari , O. Kurochkin , V. G. Nazarenko , O. D. Lavrentovich , D. Golovaty , and P. Sternberg , “Conic Sections in Ferroelectric Nematics: Experiments and Mathematical Modeling,” Physical Review Research 6 (2024): 043207, 10.1103/PhysRevResearch.6.043207.

[52] P. Sathyanarayana , V. S. R. Jampani , M. Skarabot , et al., “Viscoelasticity of Ambient‐Temperature Nematic Binary Mixtures of Bent‐Core and Rodlike Molecules,” Physical Review E 85 (2012): 011702, 10.1103/PhysRevE.85.011702. 22400578

[53] B. Kundu , R. Pratibha , and N. V. Madhusudana , “Anomalous Temperature Dependence of Elastic Constants in the Nematic Phase of Binary Mixtures Made of Rodlike and Bent‐Core Molecules,” Physical Review Letters 99 (2007): 247802, 10.1103/PhysRevLett.99.247802. 18233490

[54] S. Das , Y. L. Tang , Z. Hong , et al., “Observation of Room‐Temperature Polar Skyrmions,” Nature 568 (2019): 368, 10.1038/s41586-019-1092-8. 30996320

[55] G. Sánchez‐Santolino , V. Rouco , S. Puebla , et al., “A 2D Ferroelectric Vortex Pattern in Twisted BaTiO_3_ Freestanding Layers,” Nature 626 (2024): 529, 10.1038/s41586-023-06978-6. PMC10866709 38356067

[56] I. Luk'yanchuk , Y. Tikhonov , A. Razumnaya , and V. M. Vinokur , “Hopfions Emerge in Ferroelectrics,” Nature Communications 11 (2020): 2433, 10.1038/s41467-020-16258-w. PMC7229001 32415142

[57] I. A. Lukyanchuk , A. G. Razumnaya , S. Kondovych , Y. A. Tikhonov , B. Khesin , and V. M. Vinokur , “Topological Foundations of Ferroelectricity,” Physics Reports 1110 (2025): 1, 10.1016/j.physrep.2025.01.002.

[58] A. K. Yadav , C. T. Nelson , S. L. Hsu , et al., “Observation of Polar Vortices in Oxide Superlattices,” Nature 530 (2016): 198, 10.1038/nature16463. 26814971

[59] J. Junquera , Y. Nahas , S. Prokhorenko , et al., “Topological Phases in Polar Oxide Nanostructures,” Review of Modern Physics 95 (2023): 025001, 10.1103/RevModPhys.95.025001.

[60] C. W. Garland , “High‐Resolution Ac Calorimetry and Critical Behavior at Phase Transitions,” Thermochimica Acta 88 (1985): 127, 10.1016/0040-6031(85)85420-4.

[61] N. Sebastián , M. Lovšin , B. Berteloot , et al., “Polarization Patterning in Ferroelectric Nematic Liquids via Flexoelectric Coupling,” Nature Communications 14 (2023): 3029, 10.1038/s41467-023-38749-2. PMC10213025 37230977

