# Supplementary material for: Elasticity‐Driven Periodic Polarization Patterns in a Chiral Ferroelectric Nematic Fluid
Source: Adv Mater. 2026 Sep 23;38(62):e75002. doi: 10.1002/adma.75002 (PMC13741116; doi:10.1002/adma.75002)
Supplement: Supplementary file 1 [file ADMA-38-e75002-s005.pdf]

Supporting Information

Elasticity-Driven Periodic Polarization Patterns in Chiral Ferroelectric Nematic Fluid

Anej Sterle[1,2], Peter Medle Rupnik[1,2], Luka Cmok[1,2,3], Aitor Erkoreka,[4] Marta Lavrič[1], Natan Osterman[1,2], Calum J. Gibb[5], J. Hobbs[6], Josu Martinez-Perdiguero,[4] Richard J. Mandle[5,6], Alenka Mertelj[1], Nerea Sebastián[1*]

[1] Jožef Stefan Institute, Ljubljana, Slovenia
[2] Faculty of Mathematics and Physics, University of Ljubljana, Ljubljana, Slovenia
[3] CENN Nanocenter, Ljubljana, Slovenia
[4] Department of Physics, Faculty of Science and Technology, University of the Basque Country UPV/EHU, Bilbao, Spain
[5] School of Chemistry, University of Leeds, Leeds, UK
[6] School of Physics and Astronomy, University of Leeds, Leeds, UK

*corresponding author: nerea.sebastian@ijs.si

Contents

Supporting F7 mixture characterization

Supporting F7* mixture characterization

Supporting figures modulated instabilities

Supporting material modelling of instabilities

Videos description

Supporting F7 mixture characterization

DIO: Cr (N_F 65 °C) - N_S - 84.7 °C - N - 174 °C - Iso

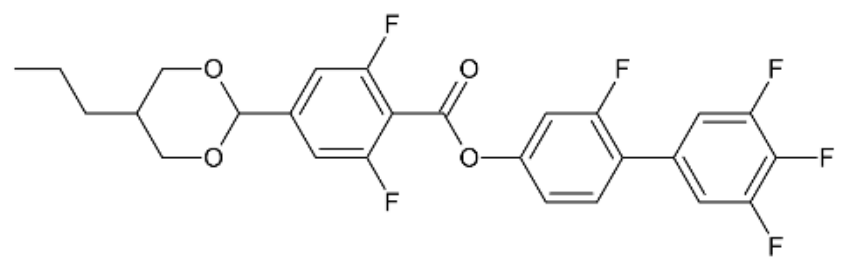

C1: Cr (SmC^H_P 90.1 °C) - SmA_F - 129.7 °C - SmA - 154.3 °C - N - 225.6 °C - Iso

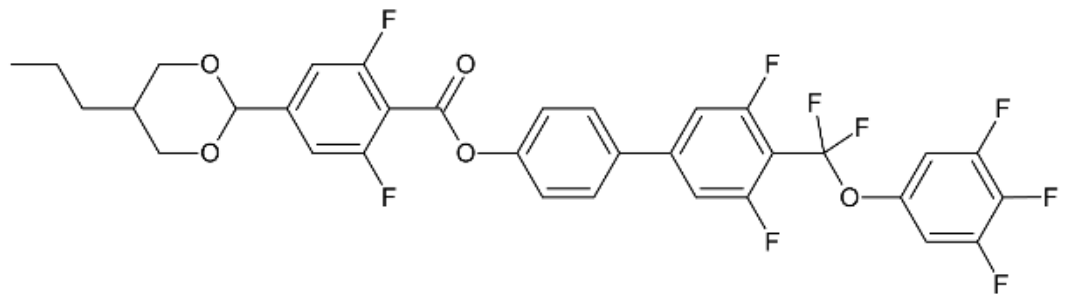

Supporting Figure 1. Molecular structure and phase sequence of DIO [1] and C1 [2].

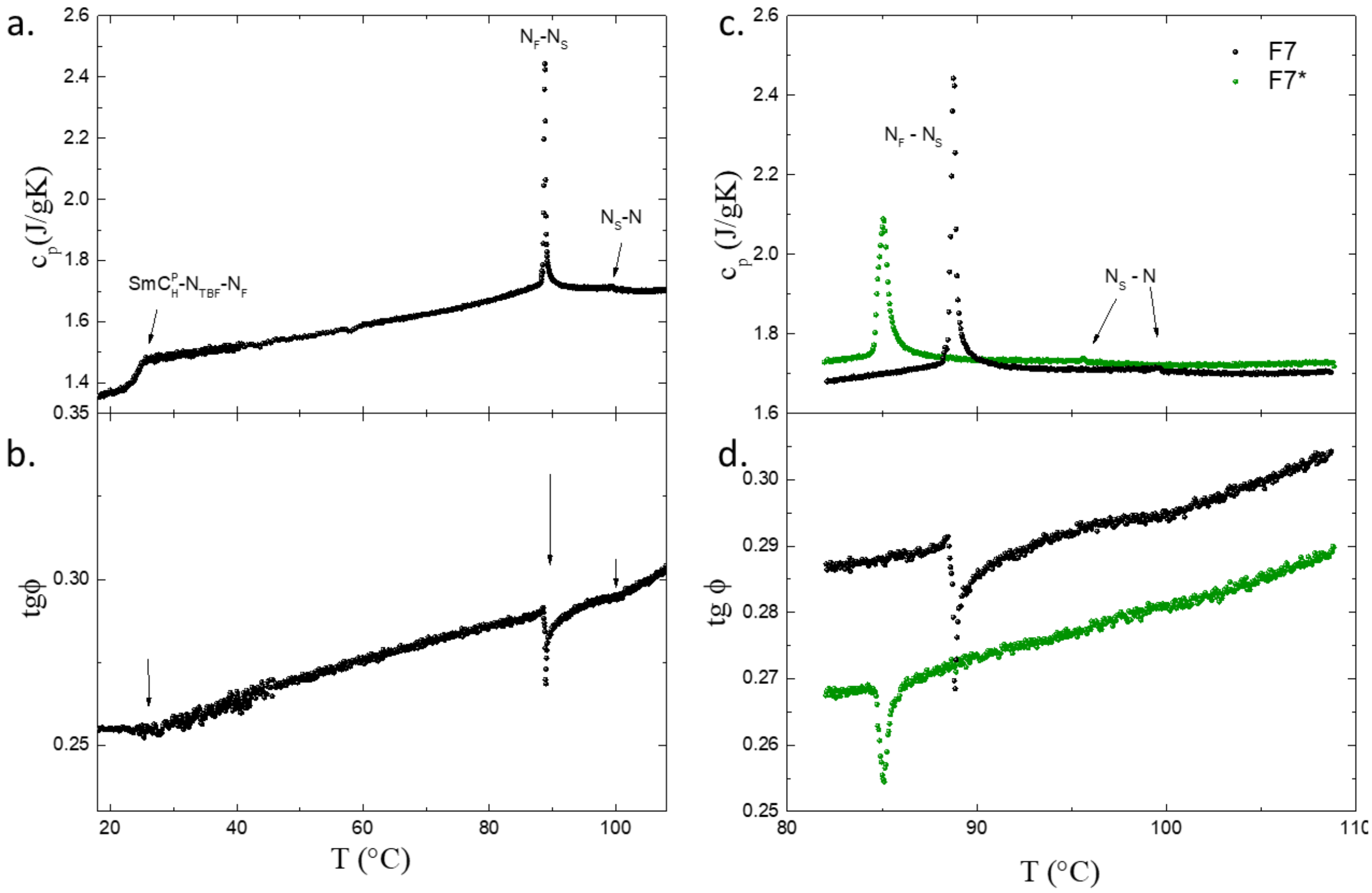

Supporting Figure 2. High resolution AC calorimetry measurements for F7 and F7*. (a) The temperature profile of the heat capacity, $c_P(T)$, for the F7 is shown upon cooling. (b) Temperature dependence of the corresponding phase shift showing negative peaks, indicating the second-order character of the transitions. (c) Temperature profile of the heat capacity, $c_P(T)$, for F7 and F7* on cooling around the N-NS-NF phase transitions. (d) Corresponding phase shift showing for F7 and F7*.

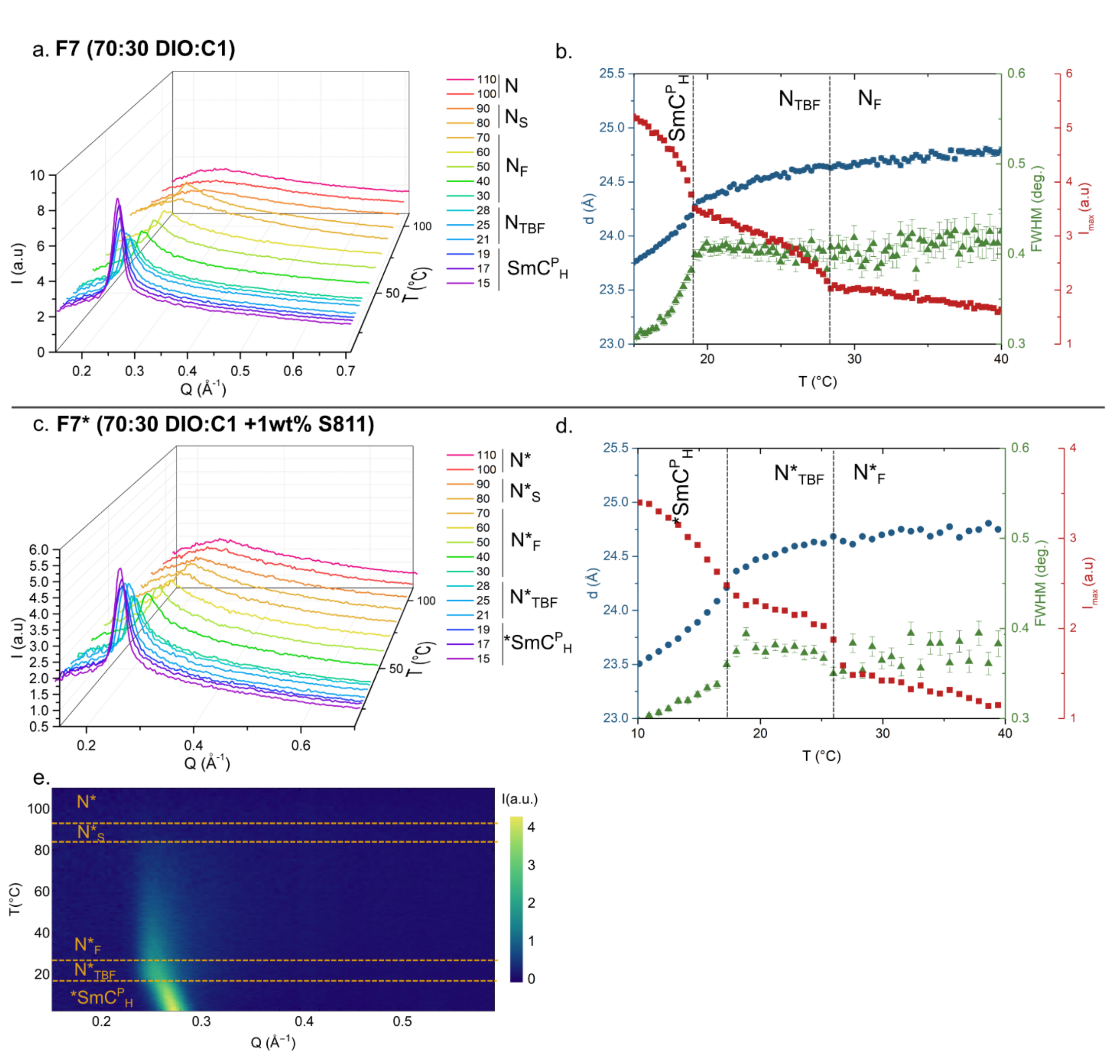

Supporting Figure 3. X-Ray experiments in F7 (a,b) and F7* samples (c,d,e). a&c) Azimuthally integrated X-ray diffractograms from 2D wide-angle non-aligned diffraction patterns at selected temperatures from the dataset in Fig. 1f. The initial broad signal characteristic of the N phase, progressively narrows in the N_F phase, showing pretransitional smectic fluctuations already throughout the N_F phase. This behaviour has previously been reported for other materials exhibiting the ferroelectric heliconical phase [3-5]. b&d) Temperature dependence of the local correlations periodicity (N_F and N_{TBF}) and layer spacing (SmC^P_H), amplitude of the scattering peak and FWHM extracted from the data presented in Fig.1.f & Figure S3.e . Peak intensity strongly marks phase transitions. At the N_F-N_{TBF} transition, a faster gradual reduction of d is observed while the FWHM plateaus. This is compatible with the growth of the twist-bend modulation without an increase in the correlation length. At the N_{TBF}-SmC^P_H transition, a fast reduction of *d* is observed as well as a strong narrowing of the signal compatible with the transition to a higher-ordered tilted smectic phase. (e) Temperature evolution of the X-ray diffractograms obtained by integration of the 2D Broad-angle XRD for an unaligned F7* sample.

SHG and polarization

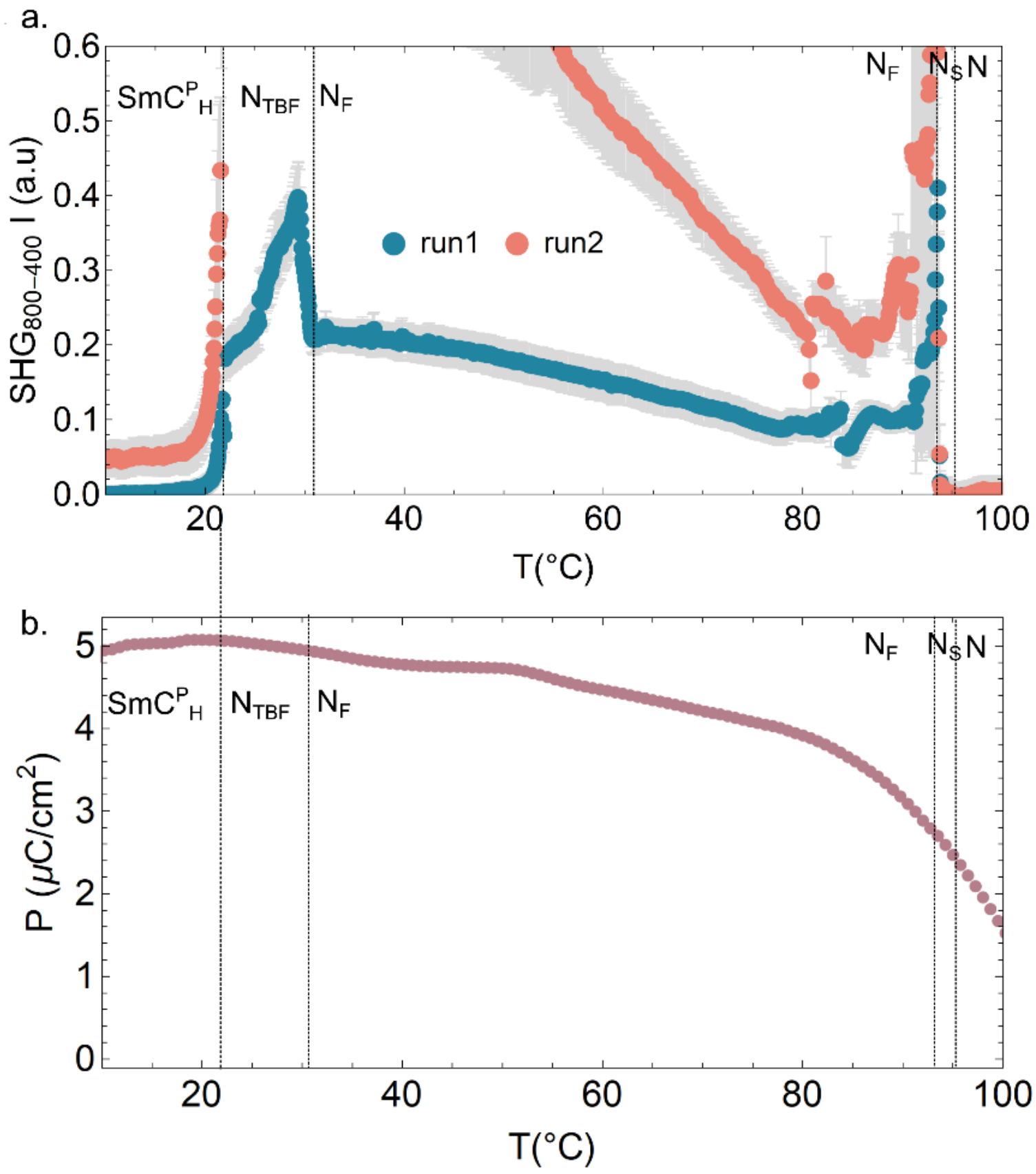

Supporting Figure 4. a) Temperature dependence of SHG signal for F7 recorded in a 5 µm parallel rubbed cell. Both runs were performed on cooling, with the fundamental beam polarization along the cell rubbing direction and no analyzer. For run2 the detector exposure time and gain were increased in order to raise the observed SHG signal in the SmC$^P{}_H$ phase above the noise level. b) Temperature dependence of spontaneous polarization in the N_F, N_{TBF} and SmC$^P{}_H$ phases. Values measured in the temperature range of the N and N_S phase correspond to induced polarization due to the high applied voltages required for polarization saturation in the N_F, N_{TBF} and SmC$^P{}_H$ phases. Polarization values are obtained via the application of a triangular wave voltage of 12 Hz and 25 Vpp amplitude in a 5 µm thick untreated cell, via the integration of the current peak measured with a 1 kOhm resistor.

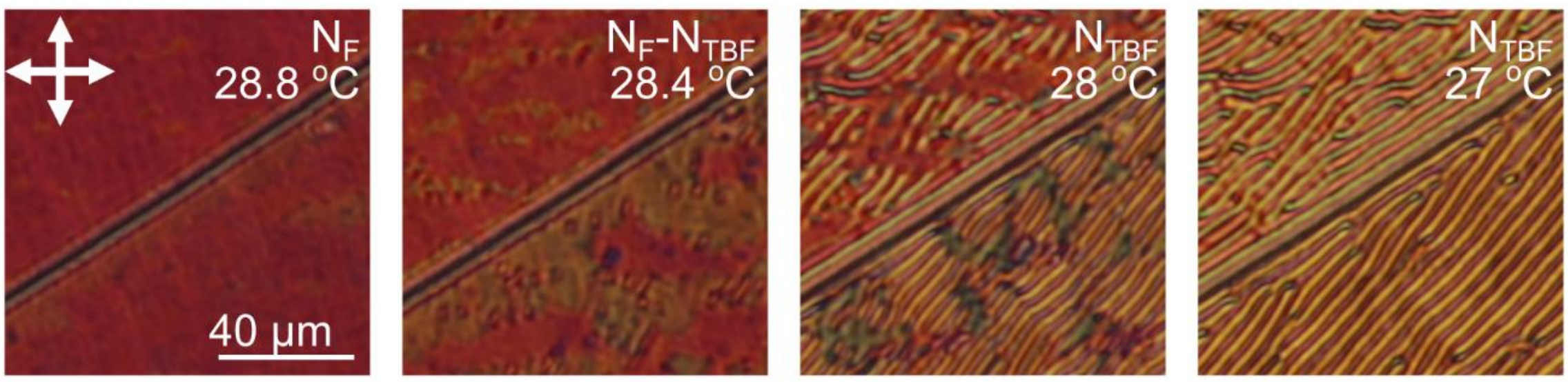

Supporting Figure 5. Polarizing optical microscopy images of F7 sample confined in a 5 µm antiparallel rubbed cell around the N_F-N_{TBF} transition showing the formation of hierarchical 3D twist in the N_{TBF} phase as described by J. Karcz et al [3] and Nishikawa et al. [4]

Supporting F7* mixture characterization

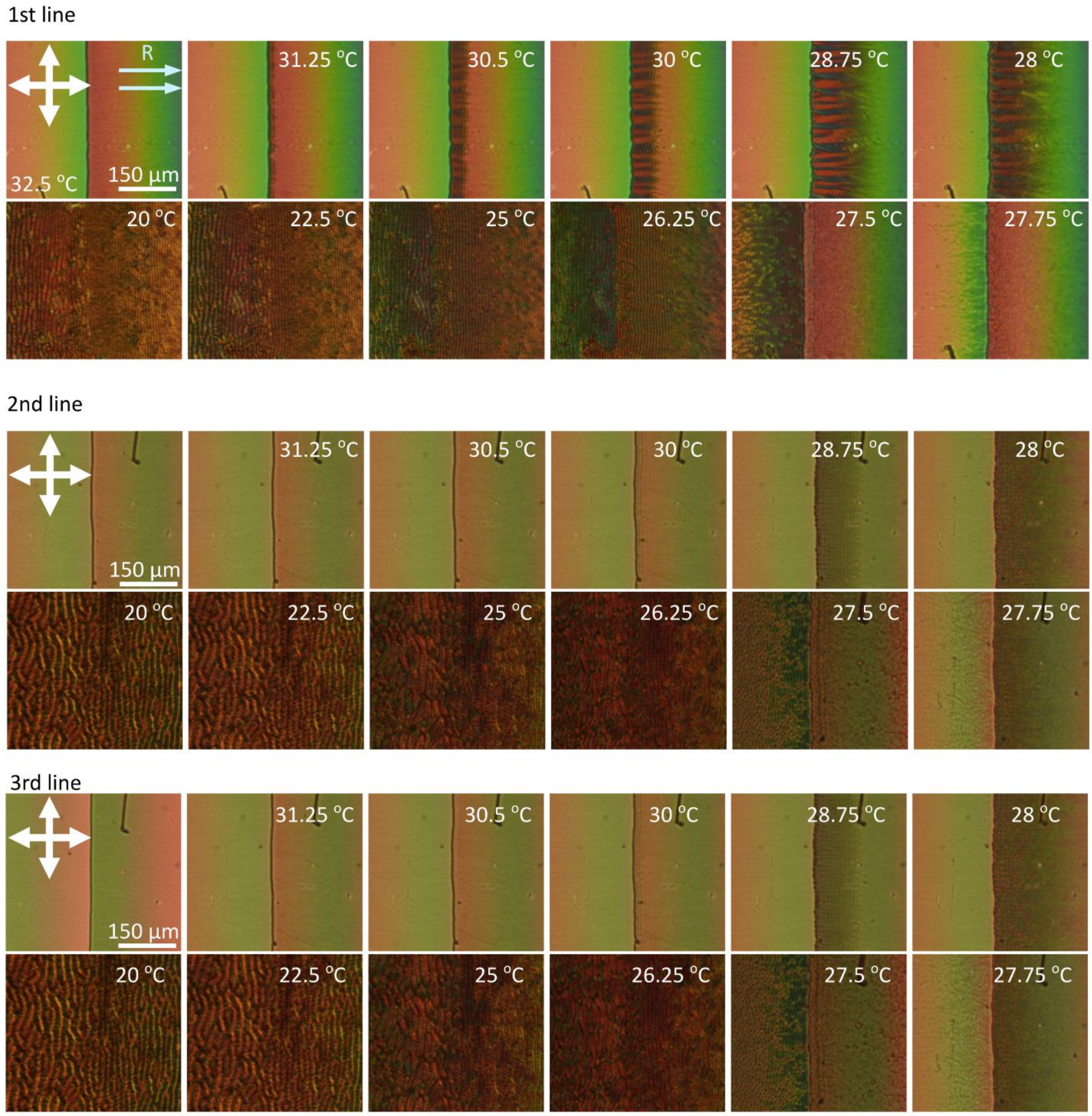

Supporting Figure 6. Zoom-ins around the 1st, 2nd and 3rd GC-lines in a wedge cell (EHC) with wedge angle $\theta = 0.68°$ and parallel rubbing along the wedge. First row of each panel corresponds to the evolution of the instabilities in the N_F* phase, while the second row corresponds to the transition to the N_{TBF}* phase and the formation of a macroscopic modulated structure.

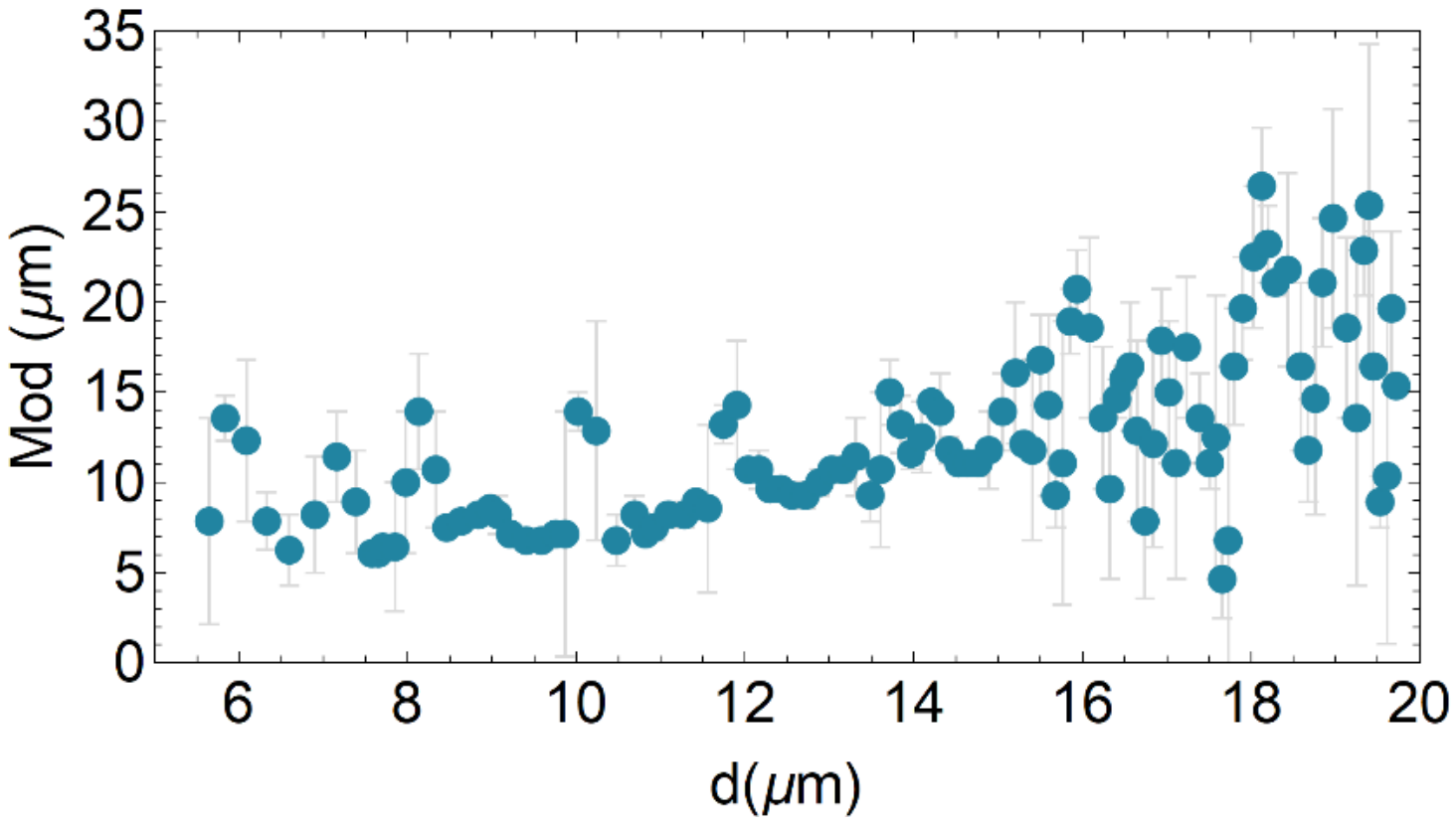

Supporting Figure 7. Macroscopic structure periodicity as a function of thickness in the N_{TBF}* phase as determined in the wedge cell (EHC).

Supporting material Modulated Instabilities

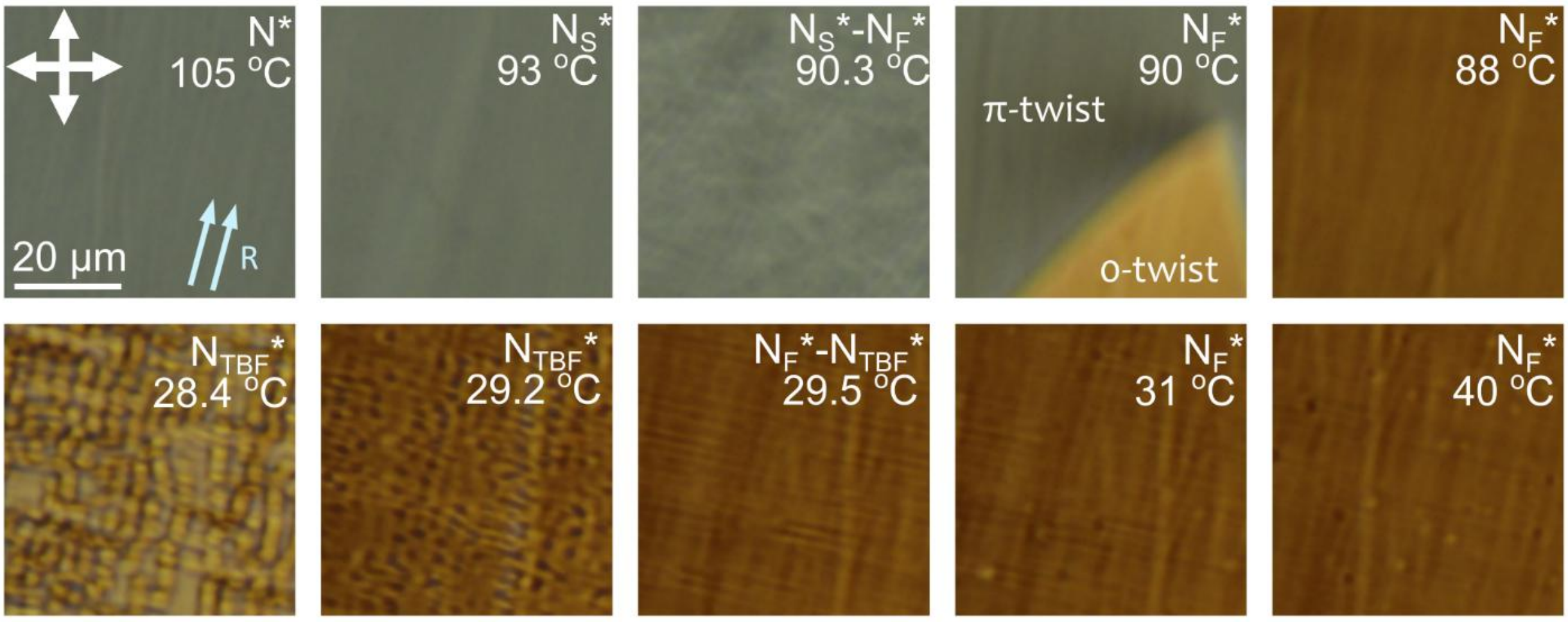

Supporting Figure 8. Polarizing optical microscopy images of F7* sample confined in a 2.2 µm parallel rubbed cell around the N_F-N_{TBF} transition.

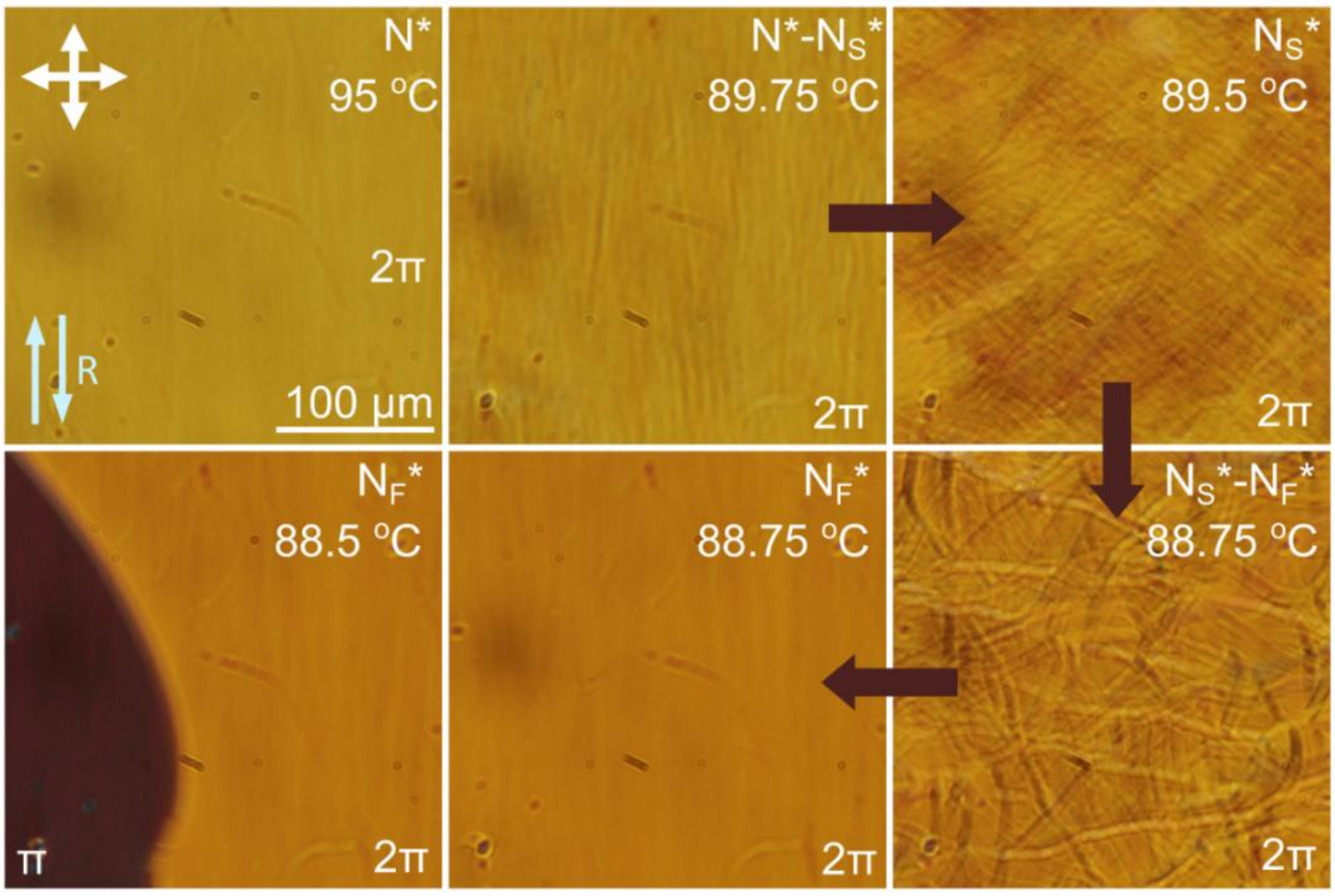

Supporting Figure 9. Formation of a π-domain for F7* in a 5.4 µm antiparallel rubbed cell on cooling from the N* phase as detected with POM. The thickness of 5.4 µm, the pitch, and the aligning conditions stabilize a hypo-twisted π-domain in the N_F* phase.

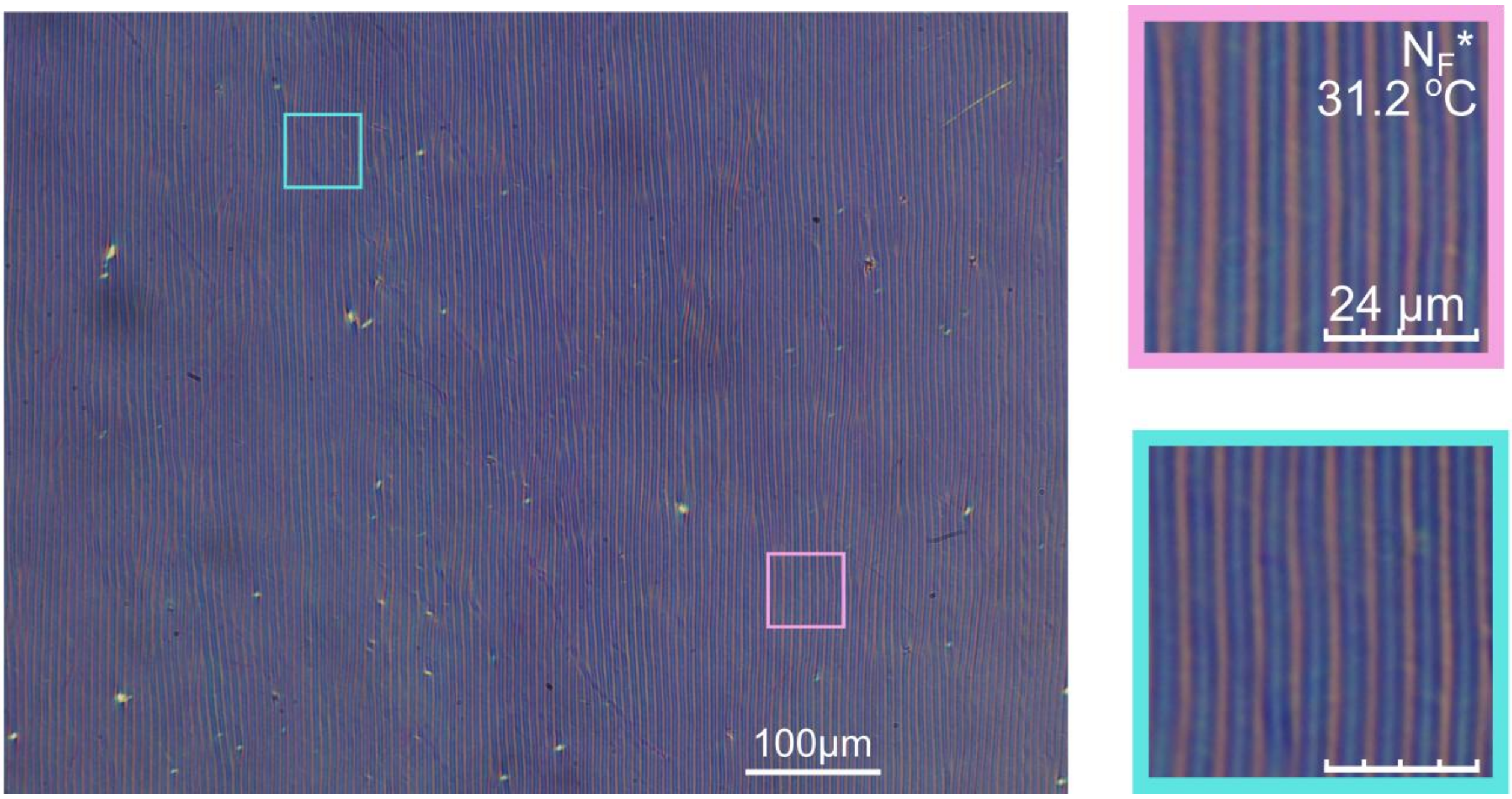

Supporting Figure 10. Overview at the onset of the instabilities for F7* in a π-twist structure obtained in a 5.4 µm antiparallel rubbed cell. Images in the right correspond to Zoom-in images of the areas highlighted in the left.

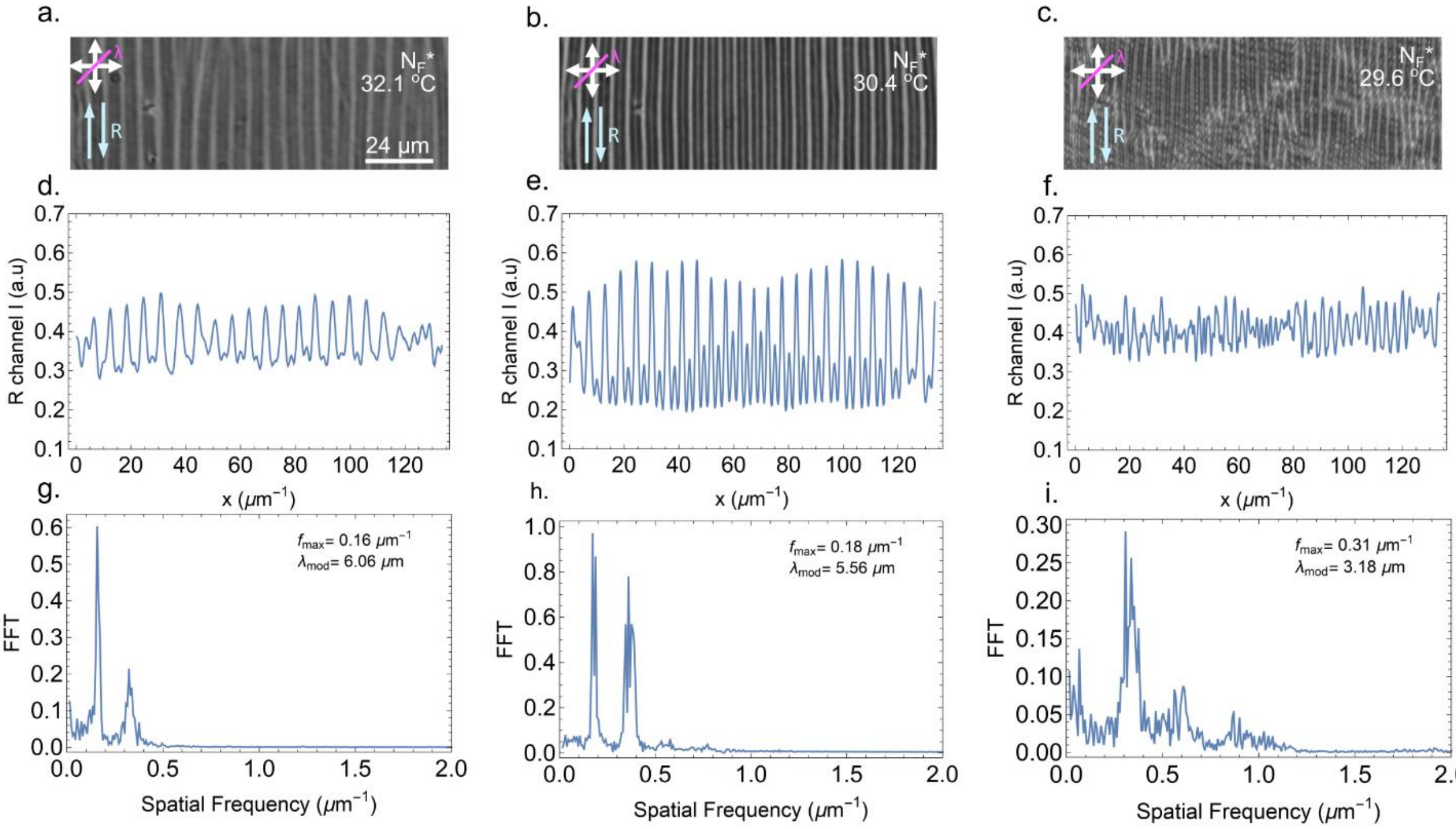

Supporting Figure 11. Example analysis of the modulated instabilities observed in the π-twist structure in a 5.4 µm antiparallel rubbed cell. (a-c) Red channel POM images at three different temperatures in the N_F* phase: 32.1 °C, 30.4 °C and 29.6 °C. (d-f) One-dimensional intensity profile as a function of *x*, obtained by averaging the pixel intensity over the entire *y* direction for the images in (a-c). (g-i) Fourier transform of the intensity profile shown in (b). The dominant peak corresponds to the characteristic modulation period of the instability and is written in the inset.

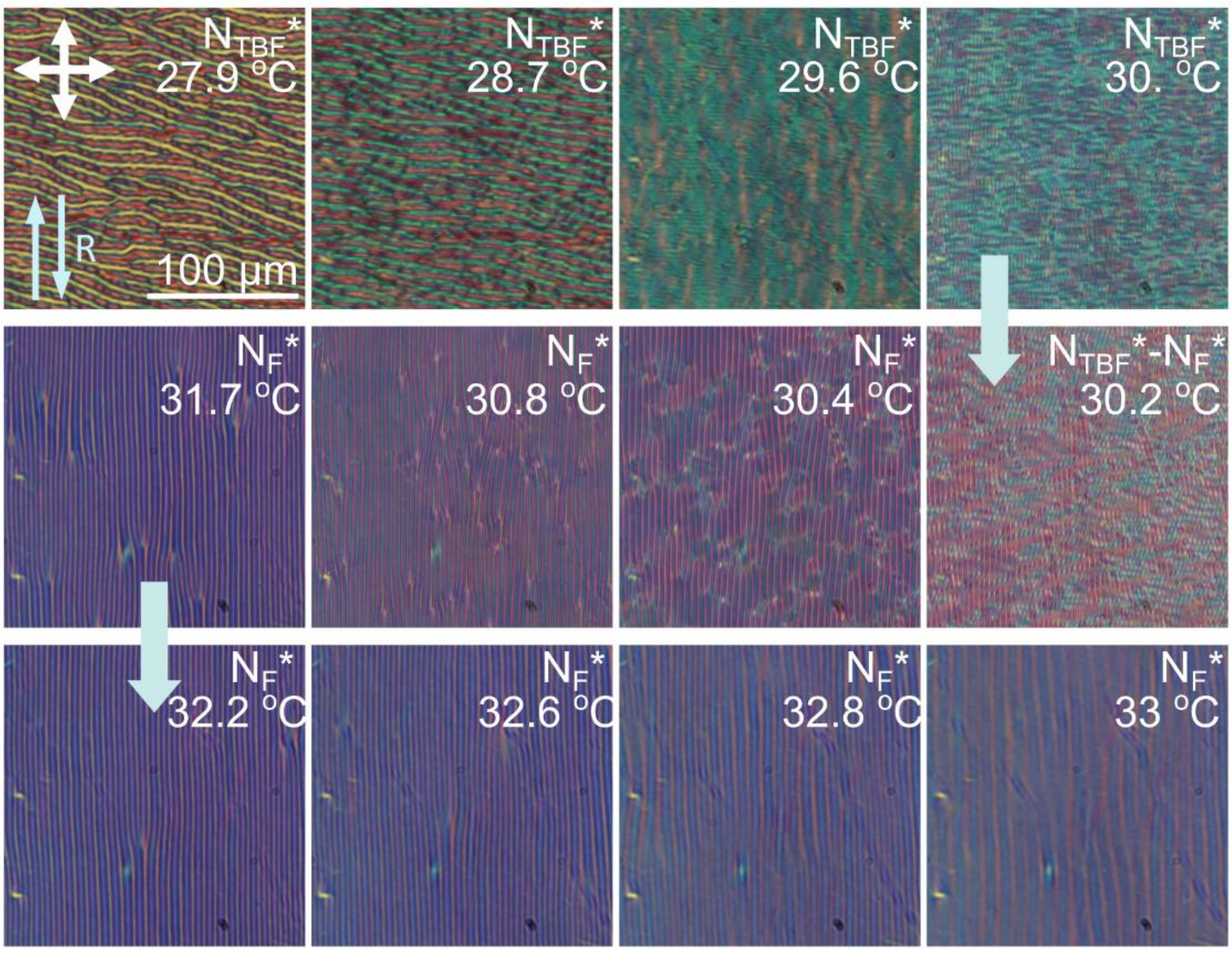

Supporting Figure 12. Polarizing optical microscopy images of the N_F*-N_{TBF}* transition on heating for F7* (F7+S811-1%) in a 5 µm antiparallel rubbed cell.

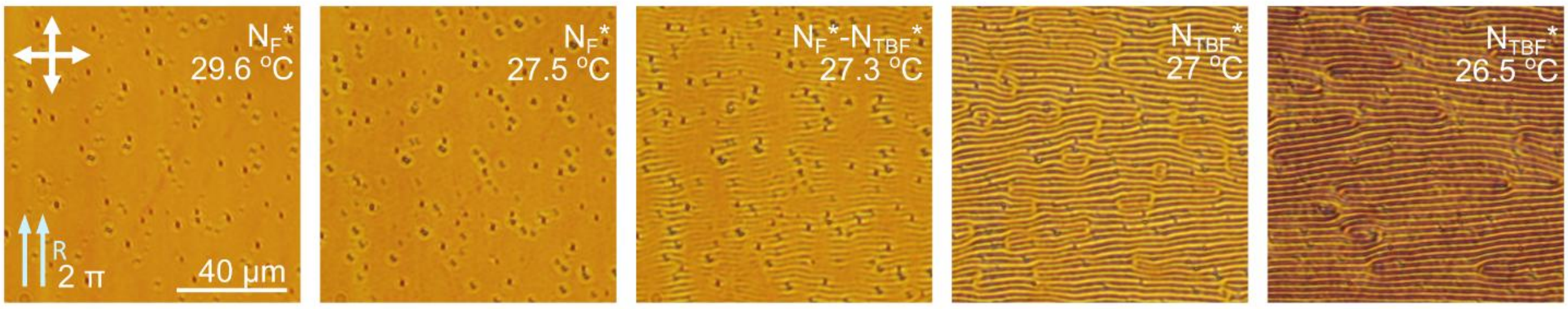

Supporting Figure 13. Polarizing optical microscopy images of the N_F*-N_{TBF}* in a 5.1 µm parallel rubbed cell. The thickness of 5.1 µm, the pitch, and the aligning conditions stabilize a hyper-twisted 2π-domain in the N_F* phase. In these conditions no instabilities are observed in the N_F*, and only a periodic modulation is observed in the N_{TBF}* compatible with the hierarchical 3D twist structures proposed by Nishikawa et al. [4].

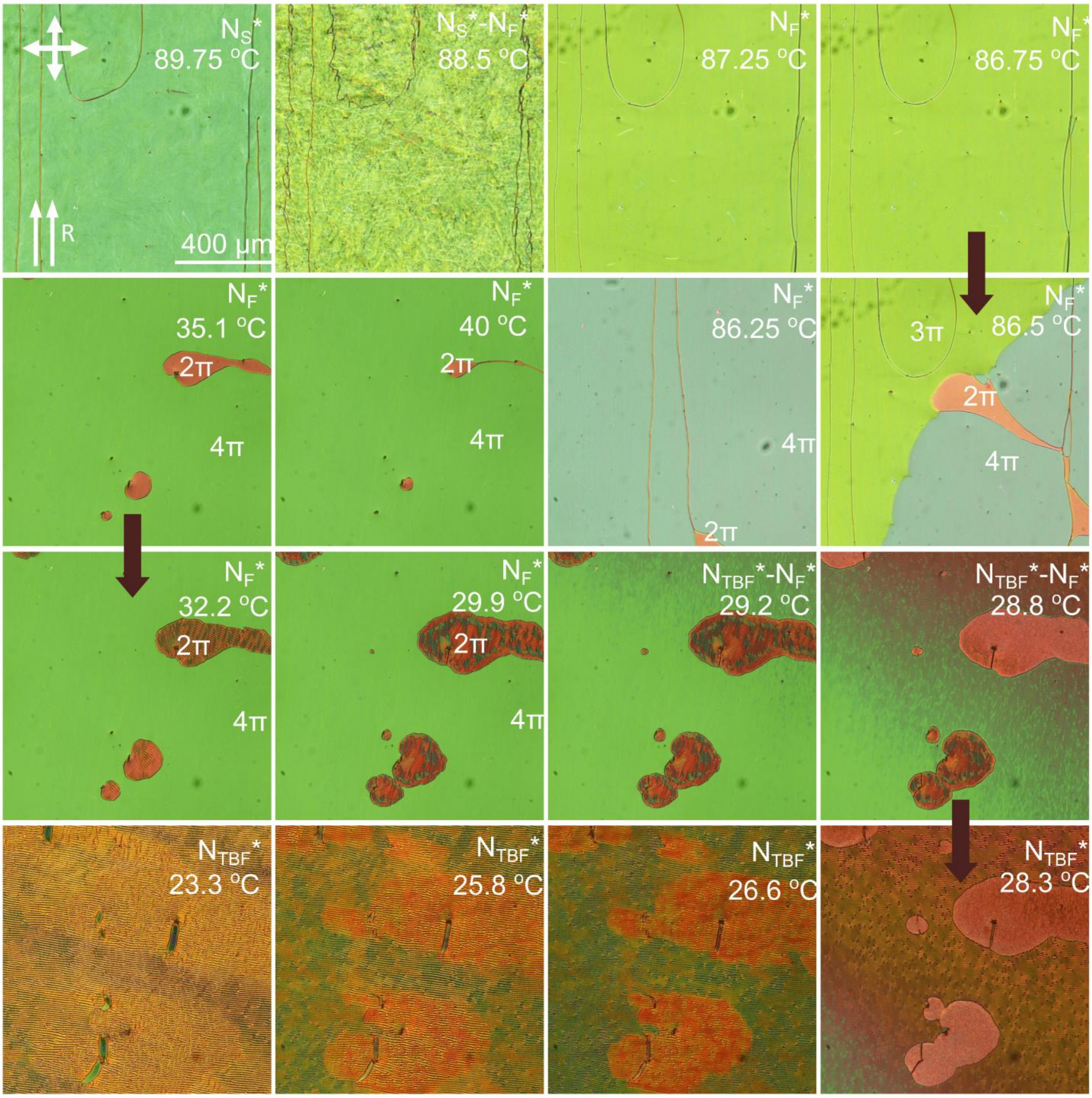

Supporting Figure 14. Formation of 2π and 4π -domains for F7* (F7+S811-1%) in a 10 µm parallel rubbed cell on cooling from the N* phase as seen by POM. The thickness, pitch and aligning conditions stabilize a hypertwisted 4π-domain in the N_F* phase. Small islands of 2π-twisted structures can also be observed. Withing these hypotwisted regions instabilities occur right before the transition to the low temperature N_{TBF}* phase. Stabilizing the sample at this temperature, as well as cycling through the N_F*-N_{TBF}* transition promotes the growth of the 2π-domains. (See Supporting Figure 15) This agrees with the observed tendency for pitch increase before the transition, which would favor 2π over 4π structures.

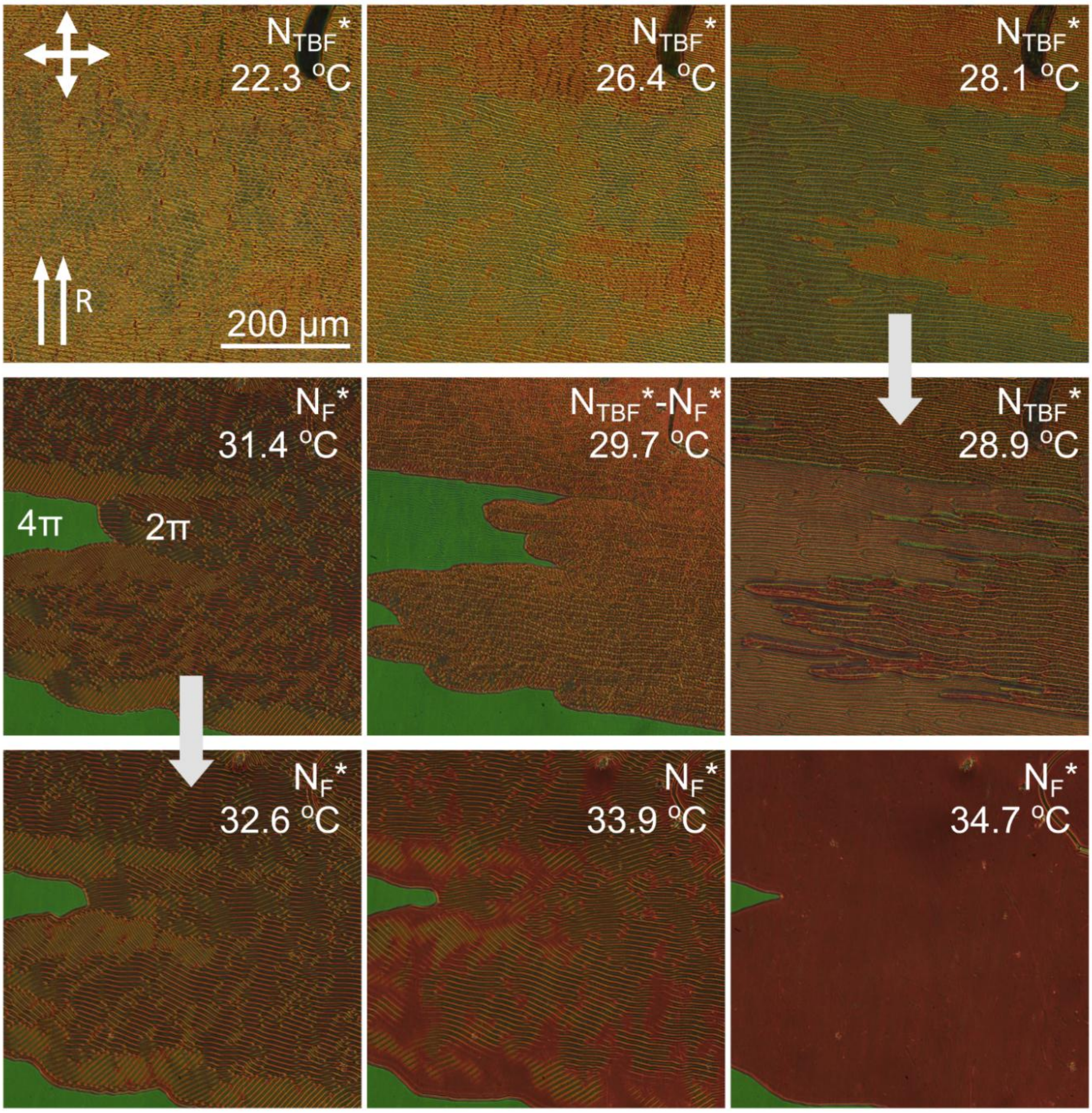

Supporting Figure 15. Polarizing optical microscopy images of the N$_F$*-N$_{TBF}$* transition on heating for F7* (F7+S811-1%) in a 10 µm parallel rubbed cell showing, as mentioned before, the stabilization of 2π-domains versus 4π-domains.

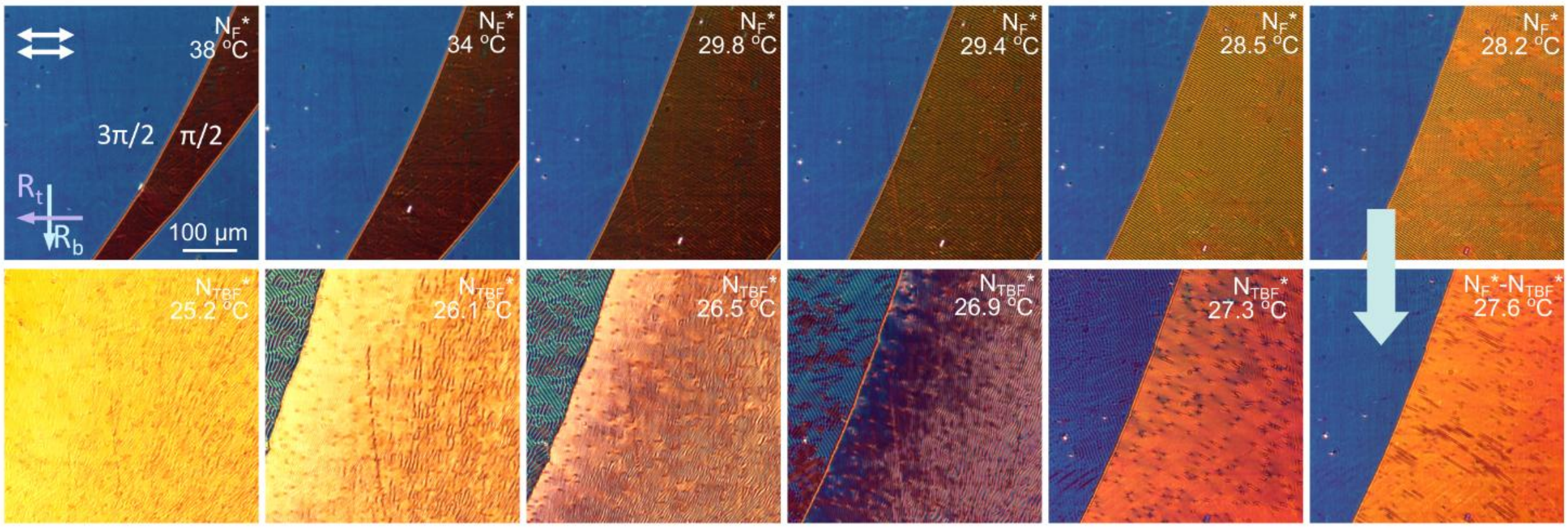

Supporting Figure 16. Polarizing optical microscopy images of the N_F*-N_{TBF}* transition on heating for F7* (F7+S811-1%) in a 5 µm left-handed 90-twisted rubbed cell, showing the different behaviour of the elastic instabilities in the π/2 and 3π/2 twisted areas. On cooling from the N* phase, 3π/2-twist structure is stabilized at high temperatures in the N_F* phase. However, at lower temperatures π/2-twsited areas start to grow due to the slight increase of the pitch.

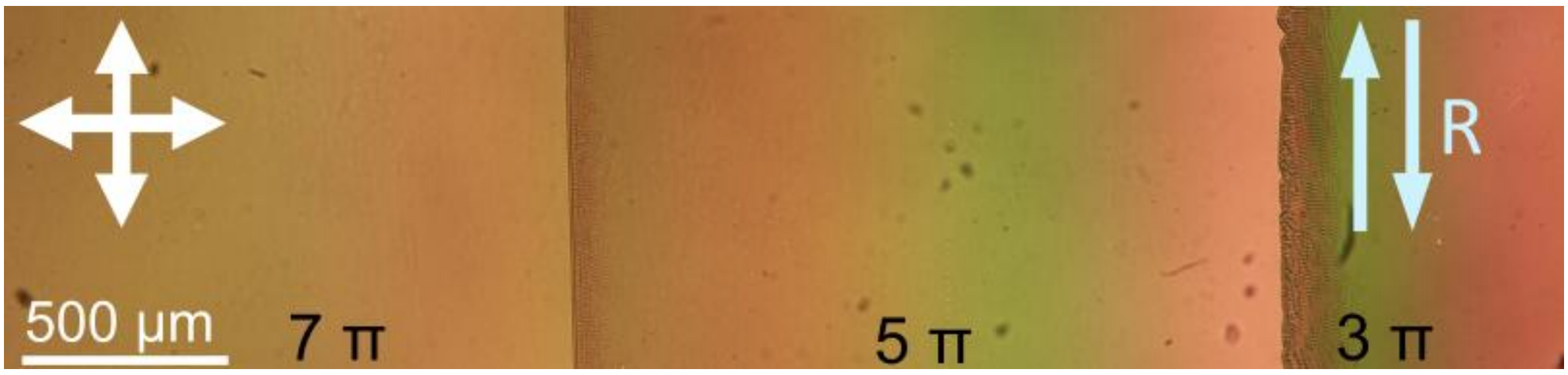

Supporting Figure 17. Overview of custom-made wedge cell with antiparallel rubbing showcasing the 3π, 5π, and 7π regions as imaged in POM in the N_F* phase at 29.7 °C. Double headed arrows indicate the position of crossed polarizers, and light-blue arrows indicate the rubbing direction in the confining cell surfaces.

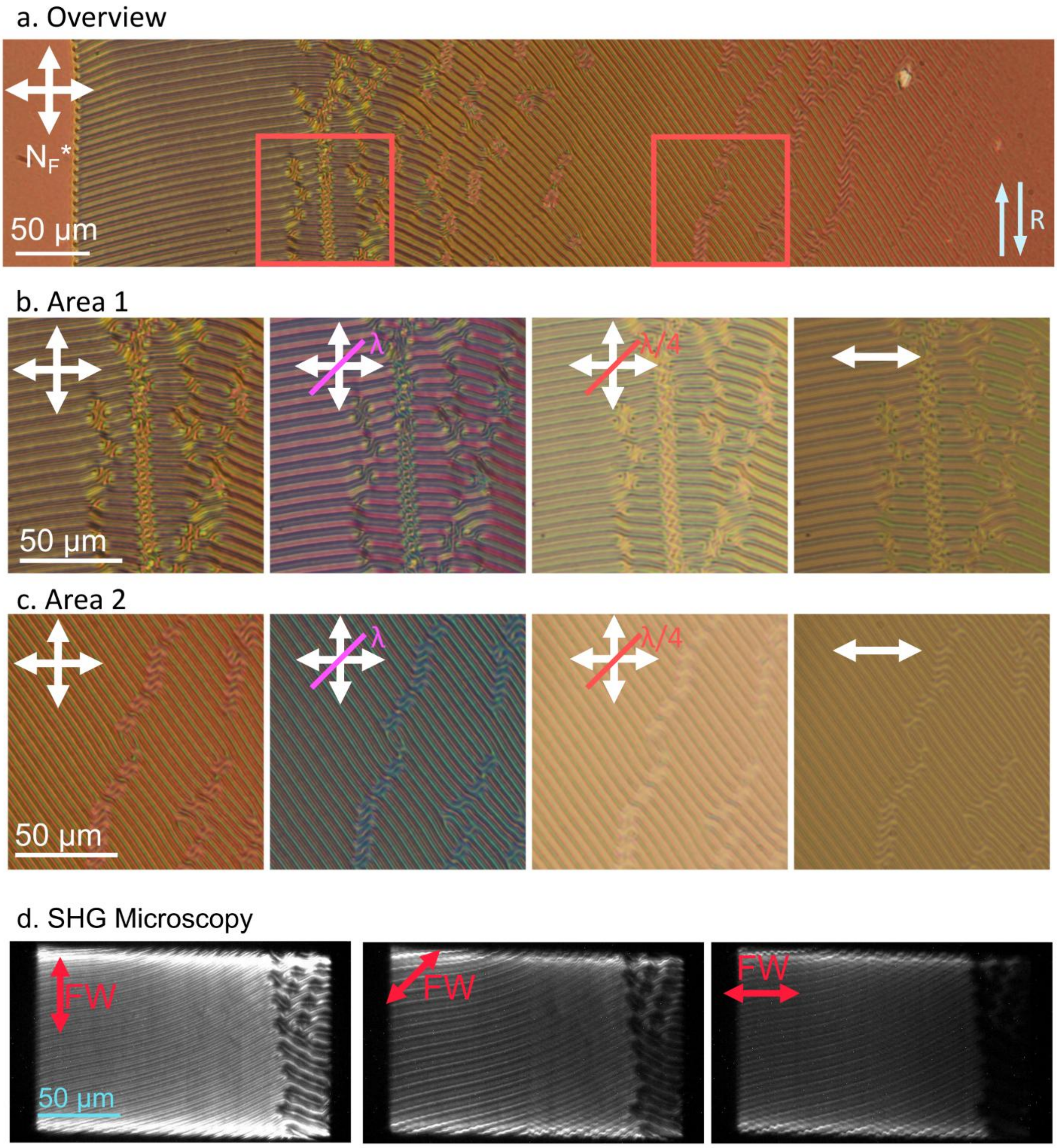

Supporting Figure 18. (a) Polarizing optical microscopy images of the 5π-3π region boundary in the N_F* phase at 29.5 °C. The top image shows an overview across different cell thicknesses. (b-c) Zoom-in images of areas marked in (a) by pink-squares at different conditions: crossed polarisers, crossed polarisers and full-lambda plate, crossed polarisers and quarter-lambda plate, and only polariser. (d) SHG-Microscopy images of the thicker 3π region section at different incoming fundamental wave polarisations and no analyser.

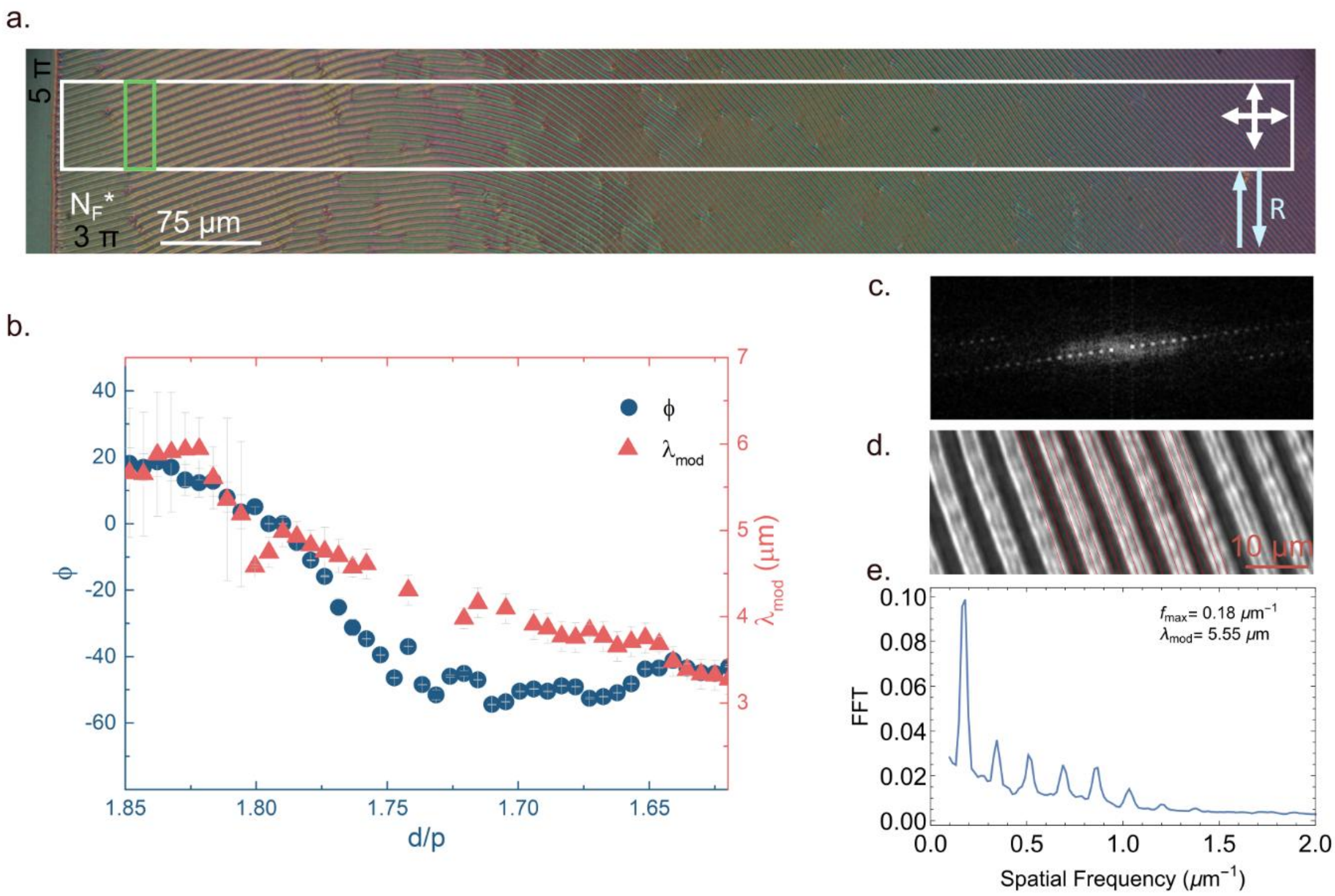

Supporting Figure 19. Analysis of the modulation wavelength and angle vs thickness in the GC 3π region in the N_F* phase at 29.0 °C. a) Shows the selected area, where the white rectangle highlights the analysed area. The analysed area was then divided into 40 subregions (green rectangle as an example) for analysis. (b) Angular direction with respect to surface rubbing and instability modulation λ_{mod} as a function of thickness, where each point pair is obtained as detailed in (c-e). (c) 2D Fourier transform of the area highlighted in green in (a). The direction of the instability modulation is calculated from the peak-position. (d) Analysed area in (c), where red lines indicate the direction detected in (c). (e) From the image in (d), a one-dimensional intensity profile is obtained by averaging the pixel intensity along the lines in (d). The plot shows the Fourier transform of the intensity profile shown, where the dominant peak corresponds to the characteristic modulation period of the instability, as plotted in (b).

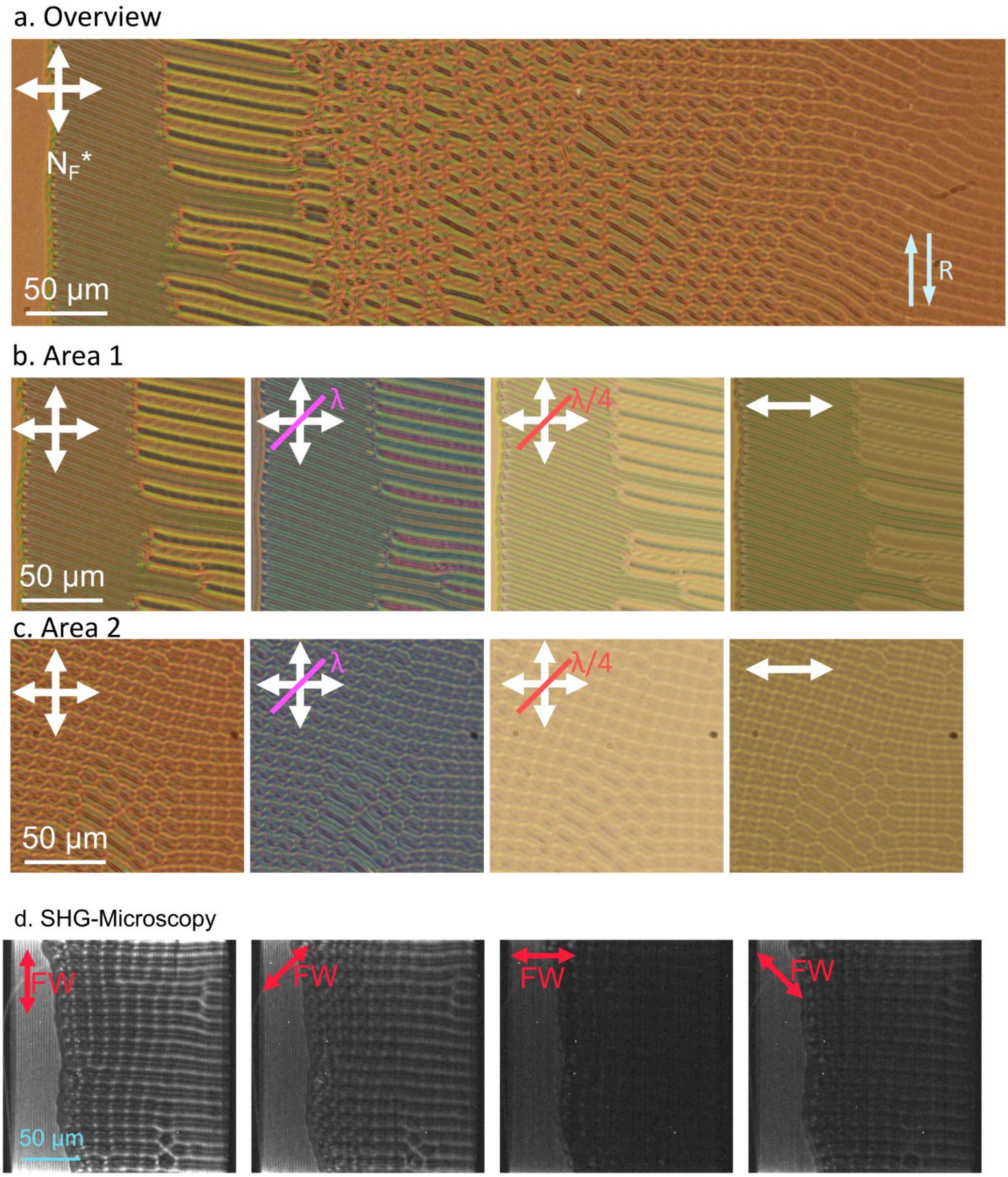

Supporting Figure 20. (a) Polarizing optical microscopy images of the 7π-5π region boundary in the N_F* phase at 29.5 °C. Top image shows an overview across different cell thicknesses. (b-c) Zoom-in images of two different areas from (a) at different conditions: crossed polarisers, crossed polarisers and full-lambda plate, crossed polarisers and quarter-lambda plate and only polariser. (d) SHG-Microscopy images of the thicker 5π region section at different incoming fundamental wave polarisations and no analyser.

Supporting material modelling of instabilities

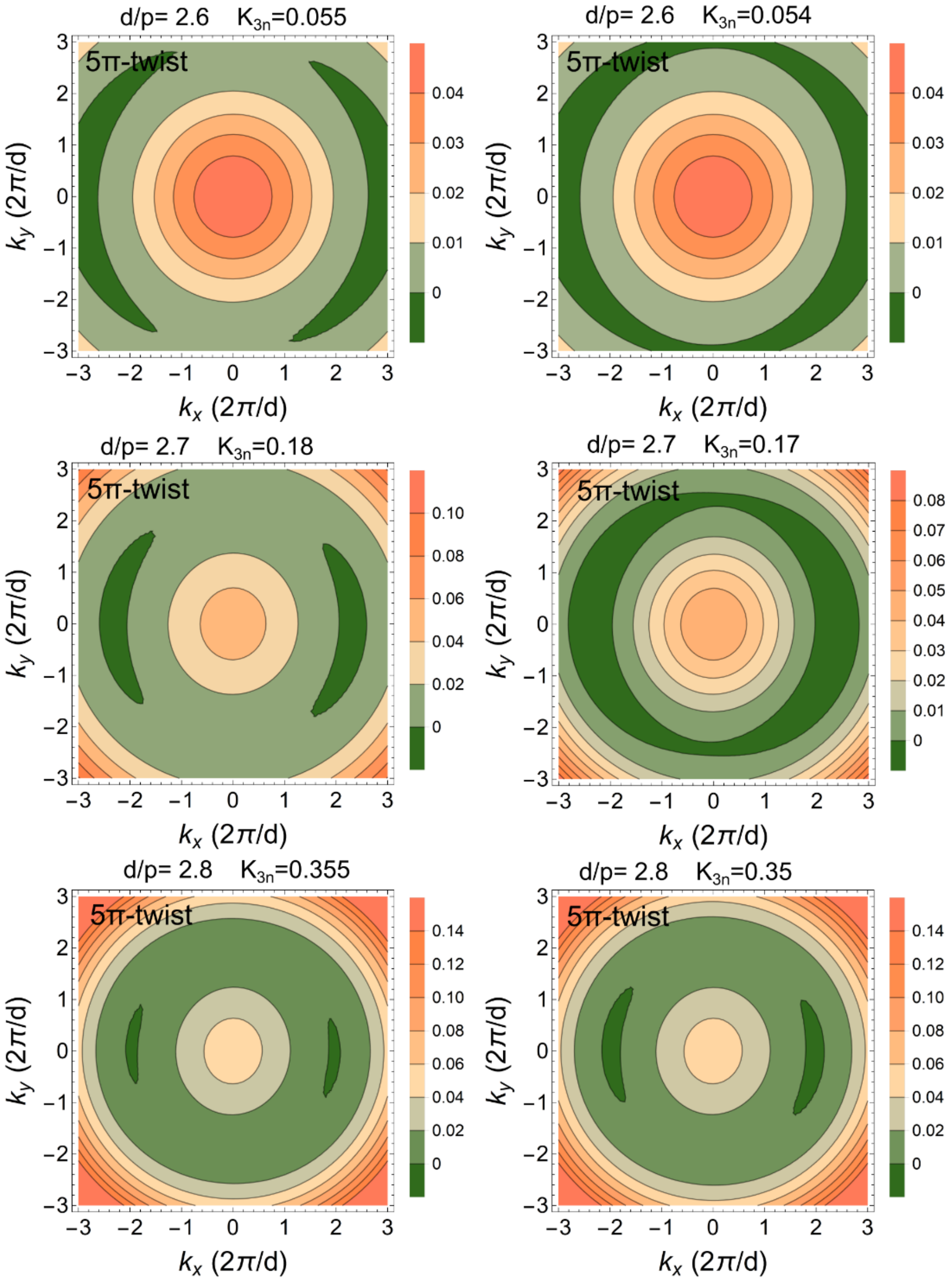

Supporting Figure 21. Two-dimensional maps of the lowest calculated eigenvalues as a function of the instability wavevector (k_x,k_y) for different confinement ratios and K_{3n}=K_3/K_2 for the 5π-twisted structures. Results show that for slightly lower K_{3n} ratios than at the onset, the dispersion promptly exhibits a shallow, round minimum, allowing multiple lattice symmetries, as experimentally observed.

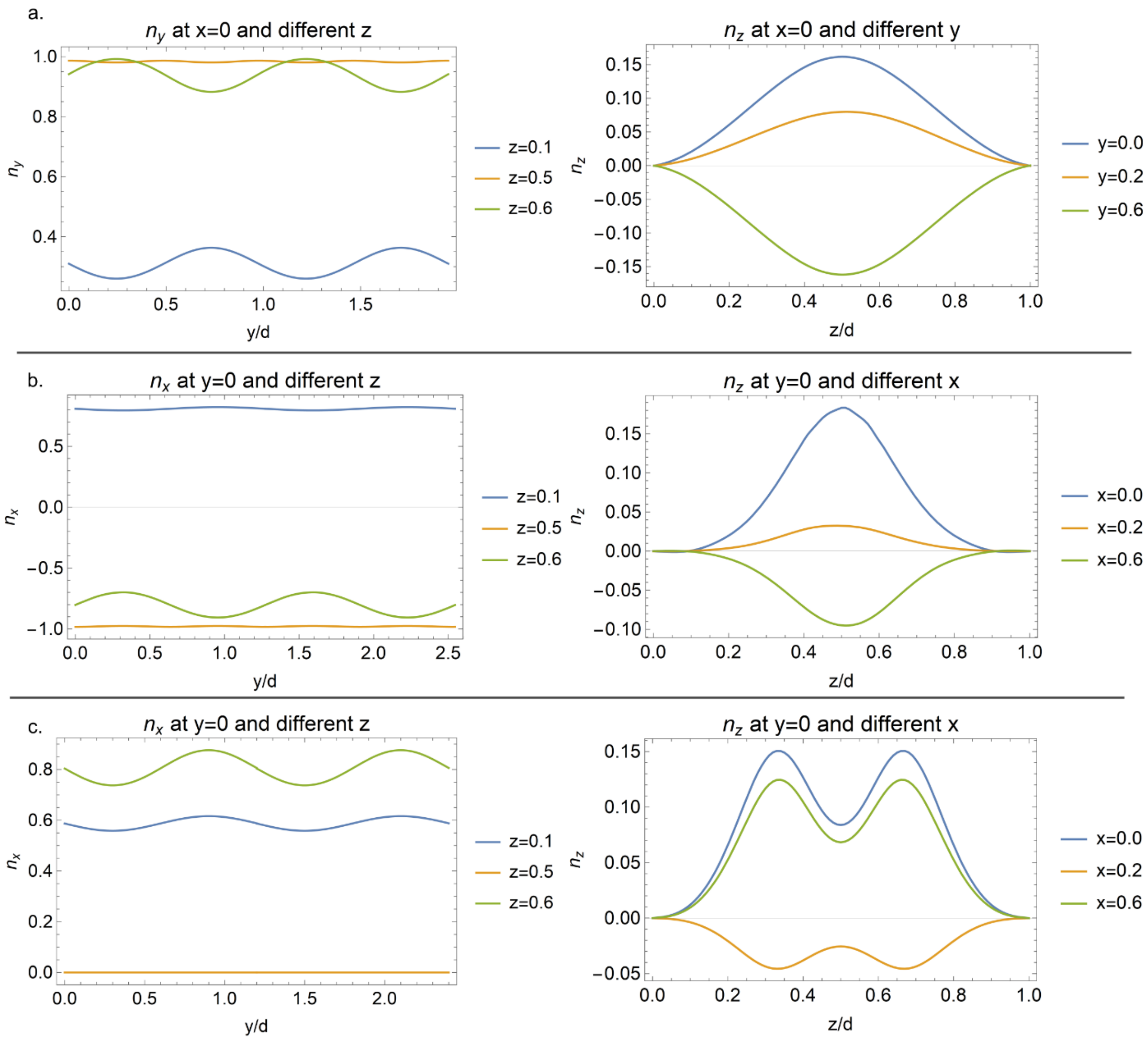

Supporting Figure 22. Profiles of the n_x, n_y and n_z components of the director field for the structures plotted in the manuscript Figure 6 for a) 1π-, b) 2π- and c) 3π-twisted structures. Left plots show the n_x(n_y) component as a function of x(y) at different positions along the cell thickness (z). The right plots show the n_z component of the director field as a function of z at different x(y) coordinates.

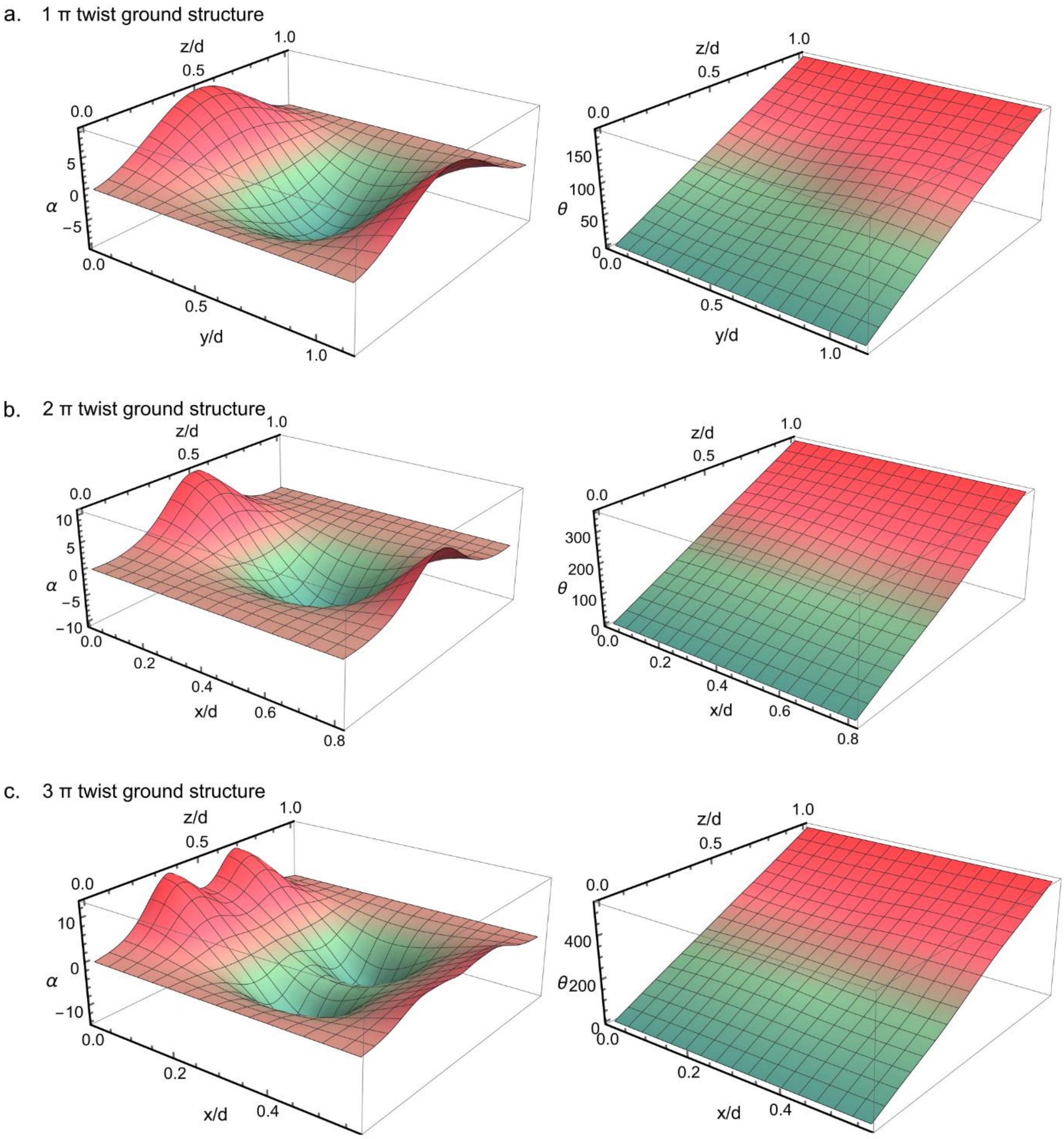

Supporting Figure 23. Spatial profiles of $\alpha(\mathbf{r})$ (left column) and $\theta(\mathbf{r})$ (right column), for (a) 1π-, (b) 2π- and (c) 3π-twisted structures. The director field is parametrized in terms of two angular variables $\theta(\mathbf{r})$ in the x-y plane and $\alpha(\mathbf{r})$ in the out of plane direction, $\mathbf{n} = (\cos\alpha(\mathbf{r})\cos\theta(\mathbf{r}), \cos\alpha(\mathbf{r})\sin\theta(\mathbf{r}), \sin\alpha(\mathbf{r}))$.

Description of Supporting Movies

Movie S1. Transition from the N_F* phase to the N_{TBF}* phase as observed by polarizing optical microscopy (POM) in a 5 µm parallel rubbed cell. The sample is placed between crossed polarizers, in vertical and horizontal directions, with the cell rubbing direction rotated around 20 degrees. The experiment was performed on cooling at 0.3 °C/min, which in the movie corresponds to 0.4 °C/s. Movie starts at 33 °C. Full movie width corresponds to 725 µm.

Movie S2. Temperature evolution of Grandjean-Cano texture in a wedge cell ($\theta = 0.68°$) with parallelly rubbed surfaces. The temperature starts at 110 °C. The experiment was performed on cooling at 0.5 °C/min, which in the movie corresponds to 2 °C/s. The sample is placed between crossed polarizers, in vertical and horizontal directions. The LC cell has parallel rubbing directions on the surface, which in the movie correspond to the horizontal direction, i.e., along the cell wedge. The thickness of the cell varies from the thinner edge (right) to the thicker (left). Full movie width corresponds to 3.5 mm.

Movie S3. Temperature evolution of the instabilities in the π-twisted structure (5.4 µm antiparallel rubbed cell) prior to the transition from the N_F* phase to the N_{TBF}* phase as observed by POM (left) and the corresponding 2D Fourier transform (Right). The experiment was performed on cooling at 0.5 °C/min. The sample is placed between crossed polarizers, in vertical and horizontal directions. The LC cell has antiparallel rubbing directions on the surface, which in the movie correspond to the vertical direction (as shown in Figure 3 in the manuscript). Full movie width corresponds to 125 µm.

Movie S4. Temperature of the instabilities in the π-twisted structure (5.2 µm antiparallel rubbed cell) prior to the transition from the N_F* phase to the N_{TBF}* phase as observed by SHG-Microscopy. The experiment was performed on cooling at 0.5 °C/min, acquiring one image every 24 seconds. The video corresponds to the temperature range from 31.5°C to 24°C, i.e. temperature rate 0.2 °C/s. Incoming 800 nm fundamental wave is polarized along cell rubbing direction, i.e. vertical in the image. No analyser is employed for image recording.

References

[1] H. Nishikawa, K. Shiroshita, H. Higuchi, Y. Okumura, Y. Haseba, S. Yamamoto, K. Sago, H. Kikuchi, *Adv. Mater.* **2017**, *29*, 1702354.

[2] C. J. Gibb, J. Hobbs, D. I. Nikolova, T. Raistrick, S. R. Berrow, A. Mertelj, N. Osterman, N. Sebastián, H. F. Gleeson, R. J. Mandle, *Nat Commun* **2024**, *15*, 5845.

[3] K. Jakub, Herman, Jakub, N. Rychłowicz, P. Kula, E. Gorecka, J. Szydlowska, P. W. Majewski, D. Pociecha, *Science* **2024**, *384*, 1096.

[4] H. Nishikawa, D. Kwaria, A. Nihonyanagi, F. Araoka, *Advanced Materials* **2026**, *38*, e13451.

[5] H. Nishikawa, D. Okada, D. Kwaria, A. Nihonyanagi, M. Kuwayama, M. Hoshino, F. Araoka, *Advanced Science* **2024**, *11*, 2405718.
